# Comparative effectiveness of Tuina therapy versus manual physical therapy for knee osteoarthritis: a randomized controlled trial

**DOI:** 10.1186/s12906-025-04850-w

**Published:** 2025-04-08

**Authors:** Peihong Ma, Luping Liu, Sina Li, Meiling Cai, Siyu Han, Zhiwen Weng, Qianji Chen, Yixuan Gao, Lingyun Zhang, Guiyun Wu, Xiaoming Yang, Yang Zhang, Duoduo Li, Changxin Liu, Ya’nan Sun, Shiyan Yan, Xiyou Wang, Changhe Yu

**Affiliations:** 1 Acupuncture and Moxibustion Department, Beijing University of Chinese Medicine, Beijing, China; 2 School of Medical Technology, Tianjin University of Traditional Chinese Medicine, Beijing, China; 3 Tuina and Pain Management Department, Dongzhimen Hospital Beijing University of Chinese Medicine, Beijing, China; 4 Pediatrics Department, Inner Mongolia Xing’an Meng People’s Hospital, Wulanhaote, China; 5 Acupuncture and Moxibustion Department, Langfang TCM Hospital, Langfang, China; 6 Acupuncture and Moxibustion Department, Luohu District Chinese Hospital, Shenzhen, China; 7 Medical Insurance Payment Department, Beijing Municipal Bureau of Medical Insurance, Beijing, China; 8 Traditional Chinese Medicine Department, Xuanwu Hospital Capital Medical University, Beijing, China

**Keywords:** Tuina therapy, Knee osteoarthritis, Physical therapy, Randomized controlled trial

## Abstract

**Background:**

Tuina therapy (Tuina) is commonly utilized for managing knee osteoarthritis (KOA), yet the available evidence is limited. This study aimed to evaluate the effectiveness of Tuina compared to widely accepted manual physical therapy (mPT) for patients with KOA.

**Methods:**

Between Oct 2019 and Oct 2021, patients with KOA (Kellgren-Lawrence score II or III) were randomly assigned in a 1:1 ratio to receive Tuina or mPT, with eight 20-min sessions over 3 weeks. Assessments were performed at baseline, week 4, 8, and 16. The primary outcome was the change of total Western Ontario and McMaster University Osteoarthritis Index (WOMAC) from baseline to week 4. Secondary outcomes included WOMAC subscales, knee pain measures, performance-based tests, quality-of-life measures, and safety assessments. Patients, evaluators, and statisticians were blinded to treatment group assignment. All main analyses were by intention-to-treat.

**Results:**

Of the 140 patients allocated to Tuina or mPT, 127 completed the treatment. There was significant intervention × time interaction observed in the WOMAC-total (F(2, 266) = 3.87, *P* = 0.02), there was no statistically significant between groups at week 4 (between-group difference: −1.00, 95%CI: −5.33 to 3.33, *P* = 0.79, Bonferroni correction). By week 8, Tuina showed significantly consistent improvement compared to mPT (between-group difference: −4.33, 95%CI: −8.34 to −0.31,* P* = 0.03, Bonferroni correction), whereas there were no statistically significant differences between groups at week 16 (between-group difference: 0.74, 95%CI: −3.67 to 5.15, *P* = 0.37, Bonferroni correction). Most secondary outcomes showed no significant between-group differences, except for the Timed Up and Go Test Time favoring mPT (0.94, 95%CI: 0.03 to 1.85, *P* = 0.04). No serious adverse events occurred. One patient in the mPT group took the medication and no patients received other therapies for KOA.

**Conclusions:**

Tuina produced beneficial effectiveness similar to mPT in treating KOA.

**Trial registration:**

NCT03966248, Registered on 29/05/2019, ClinicalTrials.gov.

**Supplementary Information:**

The online version contains supplementary material available at 10.1186/s12906-025-04850-w.

## Introduction

Knee osteoarthritis (KOA) causes pain, disability, and reduced quality of life (QoL) in adult and elderly populations, in addition to its major effect on productivity and enormous burden on the health care system [1–3]. With the combined effects of aging and increasing obesity, the prevalence of KOA continues to rise [4]. By 2032, it is estimated that the proportion of individuals aged 45 and older with diagnosed KOA will rise from 13.8% to 15.7% [3, 5]. Given the growing individual and socioeconomic burden of KOA, there is an urgent need to identify more effective treatment options to alleviate these burdens.

As a slowly progressive disease with irreversible structural change, there was no known cure for KOA*.* The goals of treatment for OA are to reduce symptoms and slow disease progression. International recommendations for the management of KOA are categorized into three areas: non-pharmacological, pharmacological, and surgical [6, 7]. Given the high cost and risks of surgical treatments, as well as the side effects of pharmacologic treatments, patients are seeking non-pharmacological alternatives. Manual therapy represents a valuable non-pharmacological treatment option for alleviating pain, stiffness, and improving physical function in KOA patients, with evidence supporting its efficacy and safety [8–11]. Currently, the focus of manual therapy for KOA has been primarily on manual physical therapy (mPT), which is recommended by several international guidelines [6, 7]. mPT involves repeated, sustained, slow, and rhythmic manual manipulations aimed at reducing pain, promoting muscle relaxation, strengthening muscles, and improving the range of motion in the affected joints.

Tuina therapy (Tuina), a form of manual therapy rooted in the theoretical framework of traditional Chinese medicine (TCM), is characterized by TCM principles, especially the theory of meridians. However, mPT differs significantly in the theoretical foundations, that is primarily concerned with the physical rehabilitation of the musculoskeletal system based on biomechanical principles. Tuina employs a wide range of technical manipulations, including squeezing, kneading, pushing, grasping and pinching, rolling, rubbing, rotating, traction, shaking and exercise [12–14], which differ from the joint mobilizations, soft tissue manipulations, and therapeutic exercise in mPT. Growing interest among researchers and clinicians explores the mechanisms behind Tuina’s clinical efficacy, which is believed to enhance blood circulation, remove blockages, realign the musculoskeletal structure, and boost the body’s natural healing abilities [12, 15, 16], while mPT often explores the therapeutic mechanisms from the principles of biomechanics and neurophysiology in the affected area.

An increasing number of clinical trials have evaluated the use of Tuina for various musculoskeletal diseases, including lower back pain and neck pain [17–20]. More recent studies have evaluated its efficacy for KOA, suggesting that Tuina may reduce joint pain and improve physical function in patients with KOA [21–23]. These studies have employed various research designs, such as crossover design [23], comparisons with pharmacological treatments [21], or combination with other therapies [22] to explore the efficacy of Tuina. Despite these studies, systematical reviews indicated that evidence supporting manual therapy for KOA remained insufficient, due to the small sample size and poor methodological quality [11, 24]. Consequently, rather than comparing Tuina with inactive treatments, this study directly compared Tuina with mPT, a similar manual therapy with established efficacy, to evaluate the effectiveness of Tuina in improving pain, physical function, and the health-related QoL for patients with KOA.

## Methods

### Study design

This was a parallel, randomized, controlled trial where recruited patients with KOA were randomized into Tuina or mPT. This study was conducted at the Tuina and Pain Management Department, Dongzhimen Hospital Affiliated to Beijing University of Chinese Medicine. Patients were recruited through posters at the outpatient and community service centers, and e-poster online (Wechat). This study was approved by the Ethics Committee of Dongzhimen Hospital Affiliated to Beijing University of Chinese Medicine (DZMEC-KY-2019-06), registered at ClinicalTrials.gov (NCT03966248, Date: 29/05/2019), and was conducted in accordance with the Declaration of Helsinki. The original protocol and amended changes were shown in Additional file 1. The reporting for the trial followed the Consolidated Standards of Reporting Trials (CONSORT) guideline [25].

### Participants

Patients with KOA were diagnosed according to the Guidelines for Diagnosis and Treatment of Osteoarthritis (2007 version) [26] and referenced to the American College of Rheumatology clinical criteria [27]. KOA patients with Kellgren-Lawrence score II or III and the pain score ≥ 4 points on the numeric rating scale (NRS) were included. The completed inclusion and exclusion criteria were presented in Additional file 2.

### Randomization and masking

Eligible patients were randomly assigned to Tuina or mPT group in a 1:1 ratio. The randomization sequence was generated using the “Proc plan” program in SAS statistical software (version 9.3). The allocation sequence was implemented using opaque, sealed envelopes sequentially numbered. To ensure concealment, the random sequence was accessed only by a designed person not involved in screening, recruitment scheduling, treatment, and assessment. After obtaining the signed consent form and confirming eligibility, the coordinator opened a randomization envelope following the sequential order. Practitioners were not blinded due to delivering different manipulations, while patients, evaluators, and statisticians were blinded to the group assignment and intervention.

### Procedures

After enrollment, each patient received unified printed booklet, including health education, self-management, and simple home exercise instruction. They were not required to perform the simple home-based exercise by themselves (see Additional file 1). In cases where patients had bilateral knees affected by KOA, the practitioner only treated the heavier one to ensure consistent time of manipulation. The descriptions of interventions are presented in Table 1 and Additional file 1. Both groups consisted of 8 20-min sessions for 3 weeks, with at least a one-day interval between each session. The patients were informed that they would receive manual therapies, without specifying whether they would receive Tuina or mPT.

**Table 1:** Brief description of Tuina therapy and manual physical therapy interventions

Interventions	Description	Time
**Tuina Therapy** **8 sessions over a 3-week period**	**Soft tissue relaxation: prone and supine position** ➢ Bladder meridian: Posterior side of the affected limb rolling, kneading, and pushing techniques ➢ Meridian of stomach, spleen, and gallbladder: anterior, medial and lateral of the affected limb rolling, kneading and pushing techniques ➢ Soft tissue relaxation of posterior knee and calf: squeezing and kneading	**Repeated 5 times** **11 min**
	**Acupoints pressure: supine position** ➢ Biguan (ST31), Futu (ST32) by thumb and middle finger of one hand, Heding (EX-LE2), Neixiyan (EX-LE4), Waixiyan (Dubi, ST35) by flexed thumb, forefinger, and middle finger of the other hand ➢ Zusanli (ST36) by the thumb of one hand and Sanyinjiao (SP6) by the forefinger of the other hand ➢ Xuehai (SP10), Liangqiu (ST34), Neixifeng (medial patellofemoral ligament), Waixifeng (lateral patellofemoral ligament), Neixiyan (EX-LE4) and Waixiyan (Dubi, ST35) by the thumbs, forefingers, and middle fingers while two pawls squeezing the knee	**One minute for each acupressure treatment;** **Repeat 3 times** **3 min**
	**Patellar manipulation: supine position** ➢ Lift the patella up to the maximum extent to leave the articular surface of femoral condyle, then slowly lower it. The operation is repeated 5 times, 0.5 min in total ➢ Press and kneads on the patella in a clockwise or counterclockwise, a total of 1 min	**1.5 min**
	**Joint passive movement: supine position** ➢ Hold the ankle and the knee joint, do maximum flexion and extension with the knee joint, swinging the knee joint quickly 10 times up and down, and then pull the knee joint once. Repeated 2 times, 0.5 min in total	**0.5 min**
	**Strength training: supine position** ➢ Straight leg raise ➢ Cycling in the air	**2 min**
	**Rest on bed**	**2 min**
**manual Physical Therapy** **8 sessions over a 3-week period**	**Stretching manual: supine position** ➢ Stretche the quadriceps, iliopsoas, hamstrings, adductor femoris, abductor femoris (iliotibial band, tensor fascia lata and gluteus muscles) and gastrocnemius	**3 min**
	**Soft tissue manual: supine position** ➢ Pressure the area along the morphology of the quadriceps, hamstrings, adductor femoris, abductor femoris and gastrocnemius, suprapatellar and peripatellar, medial and lateral capsule. Operates from superficial to the moderate depth of muscle. Repeated for 1min/time, with each area being operated 2 min.	**10 min**
	**Grade 3 or 4 Physiological movements** [28] ➢ Knee flexion ➢ Knee extension ➢ Accessory movements Grade 3: Large slow amplitude oscillatory movements performed from the middle to the end of the available joint range Grade 4: Small slow amplitude movements up to the end of the available joint play **• Femoro-tibia articulation mobilizations** Patient sits. Both knees are flexed to 90° and tracts the legs downwards towards the floor. **• Anterior–posterior/posterior-anterior accessory movement** Applying oscillatory mobilization (Grade 3 or 4) in an anterior–posterior (AP) direction on the proximal tibia. Performed with large slow amplitude oscillatory movements from the middle to the end of the available joint range with Grade 3. If needed, add tibial rotation to effectively reach the restrictive barrier with Grade 4. **• Medial–lateral/ lateral-medial Accessory movements** Medial hand on the proximal tibia and apply a lateral push while the lateral hand appliess a medial push to the proximal tibia. The manipulation is performed with large slow amplitude oscillatory movements from the middle to the end of the available joint range with Grade 3. If needed, the practitioner can add the small slow amplitude movements up to the end of the available joint play with Grade 4. **• Patellar mobilizations** The practitioner stands with their back facing the patient and places the affected leg between their upper arm and trunk, holding the distal leg with both hands. Then they slightly pull the leg down while simultaneously swinging the leg up.	**3 min**
	**Strength training: supine position** ➢ Straight leg raise ➢ Cycling in the air	**2 min**
	**Rest on bed**	**2 min**

#### Tuina group

The prescription for Tuina followed expert consensus on the regimen of Tuina for KOA [29], and the reporting for the Tuina procedure followed the STandards for Reporting Interventions in Clinical Trials of Tuina/Massage (STRICTOTM) checklist. [13].

#### Manual physical therapy group

Upon enrollment, a physical therapist evaluated the initial musculoskeletal status and range of motion. The prescription for mPT was based on the optimized treatment of the previous studies [30].

During the treatment, patients were allowed to take medication for KOA if necessary or for a long time. The use of other external therapies for KOA was prohibited. If it happened, it should all be recorded.

All practitioners were selected based on their professional background in Tuina and mPT, with each having at least 5 years of clinical experience specifically in KOA. Before the start of the study, the practitioners of Tuina or mPT underwent rigorous training to ensure adherence to standardized operating procedures of Tuina or mPT, and after training each practitioner was assessed and qualified to participate in the study. Each practitioner specialized in only one type of therapy according to their profession, training, and assessment. Patients were assigned to the same practitioner throughout the study to ensure consistency. To further minimize the risk of contamination, two kinds of therapies were conducted in separate areas to prevent exposure to different manipulations or discussions about the therapies. Practitioners were also forbidden from discussing any details about the trial with patients. The implementation procedures were reviewed regularly.

### Outcomes

The outcomes were measured at baseline, week 4, 8, and 16, which covered pain, physical function, and QoL. We implemented a one-week delay when conducting the post-treatment evaluations to exclude any local pain caused by treatment that may disturb the assessment.

#### Primary outcome

Primary outcome was the change in total Western Ontario and McMaster University Osteoarthritis Index (WOMAC) [31] from baseline to week 4. The WOMAC, a widely specific self-reported scale for evaluating KOA, contained three dimensions:pain, stiffness, and physical function (24 items, total score of 0-96, higher scores reflecting more pain, stiffness, and poorer functional impairment). Patients could rate the level of difficulty they experience for each item.

#### Secondary outcomes

The secondary outcomes encompassed pain, function, and overall QoL. In terms of pain dimension, measures included average pain of the previous week using WOMAC-pain (5 items, total score with 0-20) [31], the worst knee pain in the last 24 h using NRS (0-10, 0-“no pain” and 10-“the worst pain imaginable”). Regarding function dimension, the self-reported function included the average function of the previous week using WOMAC-stiffness (2 items, total score of 0-8), [31] WOMAC-function (17 items, total score of 0-68). [31] Performance-based functional measures, including 30 Second Time Chair Rise Test [32],Timed Up and Go Test Time (TUG) [33], and One Leg Standing Test were evaluated at baseline and week 4. Furthermore, the assessment of generic health-related QoL incorporated 12-item Short Form Health Survey (SF-12) [34] and Patient Global Assessment (PGA) on NRS (0-“A very great deal better” 10-“A very great deal worse”) from baseline to each evaluation timepoint. The Outcome Measures in Rheumatology Clinical Trials and Osteoarthritis Research Society International (OMERACT-OARSI) responder criteria were calculated to assess the response rate of treatment for KOA at week 4 [35]. The response rate criteria were shown in Additional file 3.

#### Adverse events

Adverse events were any unwanted events that caused harm to patients after the treatment. All adverse events throughout 4 weeks were managed and documented.

The credibility/expectancy of the treatment was assessed by patients with a credibility/expectancy questionnaire after the first treatment [36]. At week 4, the blinding assessment was applied to ask patients to guess the therapy they received (traditional therapy, modern therapy, or unsure).

### Statistical analysis

Based on our pilot study and the minimal clinically important difference (MCID) of WOMAC-total [30, 37], we assumed that the primary outcome (WOMAC-total at week 4) in the Tuina group was expected to have difference of 7 compared to mPT with a standard deviation for both groups of 13. Given a level of significance of 0.05, with power set at 80%, the study required 56 patients in each group. Under the 20% dropout rate assumption, 70 patients were needed for each group (a total of 140).

Data analysis was performed with intention-to-treat (ITT) set, defined as all available data from after randomization. The primary outcome was applied with repeated measures analysis of covariance, the mean difference in change from baseline to 4 weeks of WOMAC-total as the dependent variables, and intervention, time, the intervention × time interaction as independent variables, and age, sex, BMI, baseline WOMAC score, medication as the covariates. The approximate normality and Mauchly’s test of sphericity were examined. The interaction of intervention-time term was tested first. If significant, between-group differences at each time point were tested. If not significant, the intervention main effect and time effect were tested. The multiple imputation was used with 20 iterations to deal with missing data for the primary outcome.

For secondary outcomes, continuous variables with repeated measures, including the subscale of WOMAC, NRS, SF-12, and PGA, were estimated using repeated measures analysis of covariance, and 30 Second Time Chair Rise Test, TUG, and One Leg Standing Test from baseline to week 4 were applied with t-test for normal distribution, Mann-Whitney U test for nonnormal distribution. And for categorical variables-responder criteria, were compared across groups with χ^2^ tests or Fisher’s exact test. The blinding assessment used the James blinding index (range 0-1; 0, total absence of blinding; 1, complete blinding; 0.5, completely random blinding) with SAS statistical software (version 9.3).

The sensitivity analysis was performed for the primary outcome in the per-protocol set (PP), including only patients who completed the post-treatment evaluation (at week 4) without major protocol violations. Baseline characteristics of patients who provided primary outcome and those who did not were compared using t-tests or χ^2^ tests.

All analyses were performed with SPSS 24.0 (SPSS Inc). The significance level was defined as a 2-sided 0.05. The detailed statistical analysis plan was shown in Additional file 4.

## Results

### Study participants

**Fig. 1:**
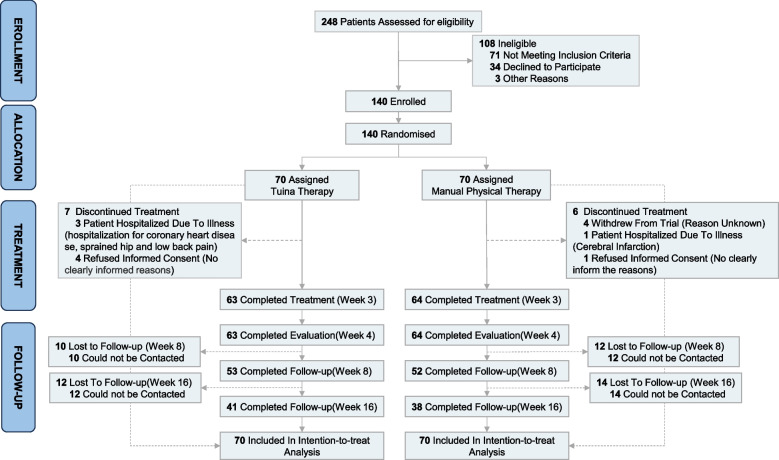
Diagram of participants flow during the study

Between Oct 2019 and Oct 2021, 248 potentially eligible participants were identified. 140 patients consented, met inclusion criteria, and were randomly assigned to two groups: 70 to the Tuina group and 70 to the mPT group. 127 (90.71%) completed the treatment at week 3 and the post-treatment evaluation at week 4, 79 (56.42%) completed the follow-up evaluation at week 16 (Fig. 1). The baseline characteristics of patients were presented in Table 2. Baseline characteristics between participants with complete and incomplete primary outcome were compared in Additional file 5.

**Table 2:** Baseline characteristics of participants

Characteristics	Tuina (*n* = 70)	mPT (*n* = 70)
**Age, mean (SD), years**	62.1 (7.6)	62.6 (8.4)
**Female, No. (%)**	54 (77.1)	55 (78.6)
**BMI, mean (SD), kg/m**^**2**^	25.1 (3.3)	24.92 (2.7)
**Race, No. (%)**		
Han	66 (94.3)	66 (94.3)
Minorities	4 (5.7)	4 (5.7)
**Duration, median (IQR), Months**	42.0 (25.0, 91.3)	42.0 (24.8, 99.0)
**Kellgren criteria, No. (%)**		
Kellgren II	39 (55.7)	38 (54.3)
Kellgren III	31 (44.3)	32 (45.7)
**Concomitant Disease, No. (%)**		
0	28 (40.0)	33 (47.1)
1	23 (32.9)	23 (32.9)
2	15 (21.4)	9 (12.9)
≥ 3	4 (5.7)	5 (7.1)
**Education, No. (%)**		
High school or less	28 (40.0)	32 (45.7)
College	39 (55.7)	32 (45.7)
Advanced degree	3 (4.3)	6 (8.6)
**Working Status, No. (%)**		
Employed	33 (47.1)	37 (52.9)
Retired	32 (45.7)	30 (42.9)
Others	5 (7.1)	3 (4.3)
**Marital Status, No. (%)**		
Married	62 (88.6)	65 (92.9)
**History of Treatment **^**a**^**, No. (%)**		
Pharmaceuticals	57 (81.4)	62 (88.6)
Interventional therapy	37 (52.9)	34 (48.6)
Physiotherapy	46 (65.7)	36 (51.4)
Others	5 (7.1)	5 (7.1)
**The Satisfaction of Previous Treatment, mean (SD)**	5.52 (2.25)	5.02 (1.69)
**WOMAC, mean (SD)**		
Total	19.67 (13.41)	18.77 (13.25)
Pain subscale	4.57 (2.73)	4.49 (2.96)
Stiffness subscale	1.74 (1.84)	1.34 (1.57)
Function subscale	13.36 (9.96)	12.94 (9.89)
**NRS, mean (SD)**	5.98 (1.55)	5.74 (1.53)
**SF-12, mean (SD)**		
Physical Health	37.56 (8.46)	37.73 (7.34)
Mental Health	50.22 (9.35)	49.67 (10.27)
**30 Second Time Chair Rise Test, mean (SD), times**	10.96 (3.75)	10.80 (3.58)
**Timed Up and Go Test Time, mean (SD), second**	11.12 (2.54)	11.48 (3.17)
**One Leg Standing Test, mean (SD), second**	19.54 (10.84)	18.58 (10.60)
**Target Knees, No. (%)**		
Left	32 (45.7)	34 (48.6)
Right	38 (54.3)	36 (51.4)
**Credibility/Expectancy Questionnaire, mean (SD)**		
Credibility questionnaire	0.25 (−0.28, 0.75)	0.25 (−0.40, 0.75)
Expectancy questionnaire	−0.01 (−1.08, 0.58)	−0.01 (−0.60, 0.88)

There were no statistically significant differences between groups at baseline for all presented variables

^a^Pharmaceuticals include painkiller, calcium, glucosamine, Chinese patent medicine, and Chinese medical decoction; interventional therapy includes intra-articular injection and lavation; physiotherapy includes exercise, massage, hot pack, acupuncture, and moxibustion

*Abbreviation*: *BMI* Body Mass Index, *WOMAC* The Western Ontario and McMaster Universities, *IQR* Interquartile Ranges

### Primary outcome

**Fig. 2:**
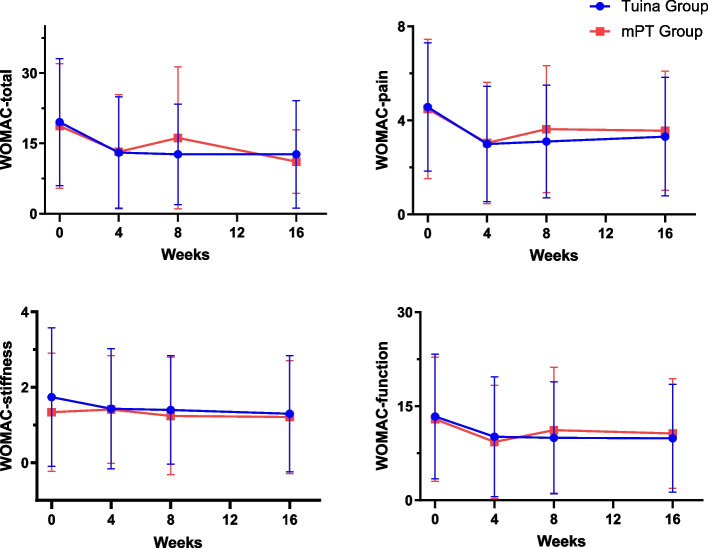
The outcome change over time. Blue line: Tuina Therapy group; Red line: manual Physical Therapy group; a: The WOMAC-total change over time; b: The WOMAC-pain change over time; c: The WOMAC-stiffness change over time; d: The WOMAC-function change over time. Abbreviation: *mPT* manual Physical Therapy, *WOMAC* Western Ontario and McMaster University Osteoarthritis Index

There was significant intervention × time interaction observed in the primary outcome ( F(2, 266) = 3.87, *P* = 0.02), which implied the treatment effect differed significantly over time. And then the between-group differences at each time point were performed to explore. At week 4, there was no statistically significant between groups (between-group difference: −1.00, 95%CI: −5.33 to 3.33, *P* = 0.79, Bonferroni correction). Similar efficacy was achieved in the PP analyses between groups (0.19, 95%CI: −4.36 to 4.74,* P* = 0.93) (Additional file 6). The comparison of primary outcome between Tuina and mPT was shown in Fig. 2.

### Secondary outcomes

As for the WOMAC-total, at week 8, Tuina showed a statistically significant greater reduction compared to the mPT group (between-group difference: −4.33, 95%CI: −8.34 to −0.31, *P* = 0.03, Bonferroni correction). However, at the follow-up period, there were no statistically significant differences between groups (between-group difference: 0.74, 95%CI: −3.67 to 5.15, *P* = 0.37, Bonferroni correction). And the change in TUG was significantly greater in the mPT group compared to the Tuina group (0.94,95% CI: 0.03 to 1.85, *P* = 0.03), and the group difference also showed clinical significance of 0.97 s [38]. Other between-group differences were not statistically significant. However, outcomes about pain (NRS, WOMAC-pain) in the Tuina at most time points showed greater improvement than mPT. Outcomes about function (WOMAC-function, WOMAC-stiffness, 30 Second Time Chair Rise Test, and One Leg Standing Test) in the Tuina also showed greater improvement than mPT, as the same with PGA; Outcomes about QoL in the mPT showed higher improvement than Tuina. The proportion of responder rate at week 4 was 29 of 70 (41.4%) in the Tuina, and 31 of 70 (44.3%) in the mPT, with no between-group difference (*P* = 0.73). The secondary outcomes were given in Table 3 and Additional file 7.

**Table 3:** Primary and secondary outcomes, differences between groups, and changes over time

Outcomes	Tuina (*n* = 70)	mPT (*n* = 70)	Between-group comparison Mean difference	Between-group *P* value ^a^
**Primary outcome**				
** WOMAC-Total**				
Baseline	19.56 (13.53)	18.73 (13.31)	0.83 (−3.66 to 5.31)	0.72
Week 4	13.05 (11.88)	13.22 (12.20)	−0.17 (−4.20 to 3.85)	0.79
Week 8	12.69 (10.74)	16.19 (15.13)	−3.50 (−7.88 to 0.89)	0.03^c^
Week 16	12.69 (11.48)	11.12 (6.76)	1.57 (−1.58 to 4.72)	0.37
Mean change from baseline-week 4 ^b^	−6.51 (−9.19 to −3.83)	−5.51 (−8.96 to −2.06)	−1.00 (−5.33 to 3.33)	0.79
Mean change from baseline-week 8	−6.87 (−9.42 to −4.32)	−2.54 (−5.69 to 0.61)	−4.33 (−8.34 to −0.31)	0.03^c^
Mean change from baseline-week 16	−6.87 (−9.95 to −3.79)	−7.61 (−10.82 to −4.41)	0.74 (−3.67 to 5.15)	0.37
**Secondary outcomes**				
** NRS**				
Baseline	5.98 (1.55)	5.74 (1.53)	0.24 (−0.28 to 0.75)	0.37
Week 4	3.56 (2.25)	3.43 (2.29)	0.13 (−0.66 to 0.93)	0.54
Week 8	4.28 (1.91)	4.77 (2.23)	−0.49 (−1.28 to 0.29)	0.74
Week 16	4.73 (1.62)	4.24 (2.13)	0.49 (−0.32 to 1.30)	0.39
Mean change from baseline-week 4	−2.33 (−2.96 to −1.71)	−2.30 (−2.95 to −1.66)	−0.03 (−0.91 to 0.86)	0.99
Mean change baseline-week 8	−1.68 (−2.24 to −1.11)	−0.86 (−1.52 to −0.21)	−0.82 (−1.67 to 0.04)	0.36
Mean change baseline-week 16	−1.19 (−1.70 to −0.67)	−1.36 (−2.01 to −0.71)	0.17 (−0.64 to 0.99)	0.85
** WOMAC-Pain**				
Baseline	4.57 (2.73)	4.49 (2.96)	0.09 (−0.87 to 1.04)	0.86
Week 4	3.00 (2.46)	3.04 (2.58)	−0.04 (−0.88 to 0.80)	0.92
Week 8	3.10 (2.40)	3.63 (2.70)	−0.53 (−1.38 to 0.33)	0.22
Week 16	3.31 (2.52)	3.56 (2.54)	−0.24 (−1.09 to 0.60)	0.57
Mean change from baseline-week 4	−1.57 (−2.21 to −0.94)	−1.44 (−2.17 to −0.71)	−0.13 (−1.09 to 0.83)	0.79
Mean change from baseline-week 8	−1.47 (−2.14 to −0.81)	−0.86 (−1.63 to −0.08)	−0.61 (−1.62 to 0.40)	0.23
Mean change from baseline-week 16	−1.26 (−1.92 to −0.59)	−0.93 (−1.69 to −0.17)	−0.33 (−1.33 to 0.67)	0.52
** WOMAC-Stiffness**				
Baseline	1.74 (1.84)	1.34 (1.57)	0.40 (−0.17 to 0.97)	0.17
Week 4	1.43 (1.60)	1.41 (1.43)	0.01 (−0.49 to 0.52)	0.16
Week 8	1.40 (1.44)	1.24 (1.56)	0.16 (−0.34 to 0.66)	0.47
Week 16	1.30 (1.54)	1.21 (1.50)	0.09 (−0.42 to 0.59)	0.70
Mean change from baseline-week 4	−0.31 (−0.64 to 0.01)	0.07 (−0.24 to 0.38)	−0.39 (−0.83 to 0.06)	0.86
Mean change from baseline-week 8	−0.34 (−0.70 to 0.02)	−0.10 (−0.49 to 0.29)	−0.24 (−0.77 to 0.28)	0.43
Mean change from baseline-week 16	−0.44 (−0.83 to −0.06)	−0.13 (−0.50 to 0.25)	−0.31 (−0.85 to 0.22)	0.27
** WOMAC-Function**				
Baseline	13.36 (9.96)	12.94 (9.89)	0.41 (−2.90 to 3.73)	0.81
Week 4	10.13 (9.56)	9.31 (9.02)	0.81 (−2.29 to 3.92)	0.61
Week 8	9.96 (8.94)	11.20 (10.04)	−1.24 (−4.42 to 1.93)	0.44
Week 16	9.89 (8.60)	10.66 (8.75)	−0.77 (−3.67 to 2.13)	0.60
Mean change from baseline-week 4	−3.23 (−5.00 to −1.46)	−3.63 (−6.18 to −1.08)	0.40 (−2.67 to 3.47)	0.80
Mean change from baseline-week 8	−3.40 (−5.12 to −1.68)	−1.74 (−4.50 to 1.01)	−1.66 (−4.88 to 1.56)	0.31
Mean change from baseline-week 16	−3.47 (−5.28 to −1.67)	−2.29 (−4.62 to 0.05)	−1.19 (−4.11 to 1.74)	0.42
** PGA**				
Week 4	7.13 (1.95)	7.10 (1.86)	0.03 (−0.64 to 0.70)	0.87
Week 8	6.38 (1.80)	6.53 (1.84)	−0.15 (−0.84 to 0.54)	0.58
Week 16	5.63 (1.92)	6.21 (1.92)	−0.58 (−1.40 to 0.24)	0.38
Mean change from week 4-8	−0.74 (−1.26 to −0.22)	−0.55 (−1.18 to 0.08)	−0.19 (−1.00 to 0.61)	0.60
Mean change from week 4-16	−1.36 (−2.06 to −0.66)	−0.71 (−1.39 to −0.04)	−0.65 (−1.61 to 0.31)	0.39
**OARSI Responder Criteria, No. (%)**				
** Responder at Week 4**	29 (41.4%)	31 (44.3%)	NA	0.73
** SF 12-Physical Health**				
Baseline	37.56 (8.46)	37.73 (7.34)	−0.16 (−2.81 to 2.48)	0.90
Week 4	41.51 (8.33)	41.75 (7.74)	−0.24 (−3.05 to 2.58)	0.88
Week 8	40.97 (8.94)	41.11 (8.94)	−0.13 (−3.51 to 3.25)	0.75
Week 16	43.50 (8.41)	44.02 (6.44)	−0.51 (−3.66 to 2.64)	0.63
Mean change from baseline-week 4	3.65 (1.30 to 6.00)	4.20 (2.02 to 6.38)	−0.55 (−3.72 to 2.62)	0.98
Mean change from baseline-week 8	2.90 (0.24 to 5.56)	3.39 (0.80 to 5.99)	−0.49 (−4.17 to 3.18)	0.84
Mean change from baseline-week 16	4.43 (1.52 to 7.34)	6.65 (3.94 to 9.37)	−2.23 (−6.16 to 1.71)	0.78
** SF 12-Mental Health**				
Baseline	50.22 (9.35)	49.67 (10.27)	0.55 (−2.74 to 3.83)	0.74
Week 4	53.48 (8.69)	53.20 (8.97)	0.28 (−2.81 to 3.37)	0.86
Week 8	53.44 (9.02)	54.63 (9.19)	−1.19 (−4.63 to 2.25)	0.44
Week 16	54.09 (9.09)	54.92 (8.16)	−0.82 (−4.45 to 2.80)	0.58
Mean change from baseline-week 4	2.82 (0.43 to 5.21)	2.96 (0.53 to 5.39)	−0.14 (−3.52 to 3.24)	0.87
Mean change from baseline-week 8	2.41 (−0.59 to 5.42)	4.27 (0.99 to 7.55)	−1.86 (−6.25 to 2.54)	0.62
Mean change from baseline-week 16	2.95 (−0.44 to 6.34)	3.71 (0.52 to 6.91)	−0.76 (−5.37 to 3.84)	0.52
** 30 Second Time Chair Rise Test, times**				
Baseline	10.96 (3.75)	10.80 (3.58)	0.16 (−1.07 to 1.38)	0.80
Week 4	13.56 (3.97)	13.11 (3.47)	0.45 (−0.86 to 1.76)	0.50
Mean change from baseline-week 4	2.37 (1.67 to 3.06)	2.38 (1.40 to 3.35)	−0.01 (−1.19 to 1.17)	0.99
** Timed Up and Go Test Time, second**				
Baseline	11.12 (2.54)	11.48 (3.17)	−0.36 (−1.32 to 0.60)	0.46
Week 4	9.94 (2.02)	9.60 (1.99)	0.35 (−0.36 to 1.05)	0.33
Mean change from baseline-week 4	−1.06 (−1.65 to −0.47)	−2.00 (−2.70 to −1.30)	0.94 (0.03 to 1.85)	0.04^c^
**One Leg Standing Test, second**				
Baseline	19.54 (10.84)	18.58 (10.60)	0.95 (−2.63 to 4.54)	0.60
Week 4	24.63 (8.11)	22.20 (9.60)	2.42 (−0.70 to 5.54)	0.13
Mean change from baseline-week 4	5.21 (3.12 to 7.31)	4.08 (2.05 to 6.11)	1.14 (−1.75 to 4.02)	0.44
** Adverse Events, No. (%)**				
Severe adverse events	0	0	NA	-
Local pain after treatment	3 (4.3)	4 (5.7)	NA	1.00
** Rescue Medicine, No. (%)**				
Week 4	0	1 (1.4)	NA	1.00

^a^: Adjustment for multiple comparisons by Bonferroni. WOMAC-total was significantly influenced by the interaction of treatment and time. And the WOMAC subscales, PGA, SF-PH, and SF-MH were not significantly influenced by the interaction of treatment and time. Between-group *P*-value tests for a significant between-group difference at each timepoint and the change score from each timepoint to baseline

^b^: The change of WOMAC-total from baseline to week 4 was the primary outcome

^c^ : indicated the differences between groups

Values are mean (SD) unless stated otherwise. Mean change presented with mean (95% CI)

*Abbreviation*: *CI* Confidence Intervals, *mPT* manual Physical Therapy, *NA* Not applicable, *NRS* Numeric Rating Scale, *OARSI* OsteoArthritis Research Society International, *PGA* Patient Global Assessment, *SF 12* 12-item Short Form Health Survey, *WOMAC* The Western Ontario and McMaster Universities

### Adverse event

Three cases in the Tuina and 4 cases in the mPT group reported local pain after the treatment, respectively, with no between-group difference ( *P* > 0.99) (Table 3). One patient in the mPT group took the medication and no patients received other therapies for KOA.

One hundred twenty-seven patients responded to the blinding assessment at week 4. 57.48% (73/127) were unsure of their treatment allocation, 30.16% (19/63) in the Tuina, and 21.88% (14/64) in the mPT believed that they received Tuina therapy. The James blinding index indicated low risk of unblinding (0.75, 95%CI: 0.68 to 0.81) (Additional file 8).

## Discussion

This randomized clinical trial provided evidence of the comparative effectiveness of Tuina and mPT for KOA. No significant differences were observed between groups for the primary outcome (WOMAC-total) at week 4. However, Tuina demonstrated a greater reduction through week 8 compared to mPT.

Until now, this is the first trial to select recommended mPT as the control to validate the effectiveness of Tuina and assess whether it performs at least as well as the established efficacy. The results revealed no significant difference between Tuina and mPT in most outcomes by 4 weeks, which aligns with previous research comparing similar manual manipulations [20, 39]. Both Tuina and mPT share core therapeutic goals - achieve pain relief, muscle relaxation, increased joint mobility, and muscle strength-yet they achieve these objectives through distinct mechanisms (see Additional file 9). Tuina mainly involves passive manipulation, such as pressing, pushing, and kneading the multiple meridians across the knee joint to relax the tissues, alongside low-amplitude knee joint mobilization [20]. Similarly, mPT focuses more on the biomechanical aspects of rehabilitation, and involves passive and active components aimed at restoring joint function, muscle strength, and flexibility. The similar techniques used in both interventions may contribute to their comparable efficacy. Acupressure or mechanical manipulations may regulate the proliferation and differentiation of articular cartilage cells to relieve articular cartilage degeneration [23], and it could enhance local microcirculation and reduce levels of inflammatory cytokines through rubbing and kneading. Similarly, mPT achieves similar outcomes through soft tissue techniques and joint mobilizations [40, 41]. Both Tuina and mPT restore optimal joint function, reducing stiffness and improving mobility.

The distinctive mechanisms and theoretical frameworks of Tuina and mPT may explain the partially divergent outcomes observed. The mPT regimen emphasizes active participation, particularly through mobilizations and exercise. These activities have a direct impact on functional mobility and muscle strength. This may explain the greater improvements in the performance-based outcome-TUG test when compared to Tuina. Tuina incorporates acupressure and meridian stimulation to promote meridian flow and restore balance within the body [13, 42], potentially contributing to sustained improvements in joint health and overall wellness. This may explain the sustained benefits observed in the Tuina group, with greater reductions in WOMAC-total through week 8 compared to mPT. Our results suggest that patients have a broader range of treatment options, enabling them to choose an approach tailored to therapeutic needs and their preferences. Furthermore, integrating both manual therapies allows practitioners to better meet each patient’s diverse requirements, improving both immediate and sustained effectiveness.

The effects of Tuina for KOA has been evaluated in a limited number of studies, with varied outcomes. In our study, Tuina demonstrated a large standardized effect size (Cohen d, ES) of 0.51, calculated for the within-group changes as mean change from baseline to week 4 divided by pooled SD (baseline SD + follow-up SD) / 2) [43, 44]. Additionally, the relative change rate [(Current value−Baseline value) / Baseline value) *100%] of 33%. However, both of these measures were lower than those reported in several previous trials. For instance, a crossover trial comparing Tuina with health education for KOA found an ES of 2.15 and a change rate of 46.46% [23]. Other studies examining the combination of Tuina with Chinese herbal treatments, or Swedish massage, also reported higher ES and change rates than our study [10, 22]. Several factors could explain these discrepancies across these studies. The dosage and treatment frequency appear to be important factors [22]. Swedish massage interventions mainly consisted of an initial course with weekly 60-min massages over 8-week, followed by long-term biweekly maintenance for 52 weeks, which far exceeded the dosage of our study. mPT in our study with relatively shorter treatment duration and frequency demonstrated a lower ES compared to previous meta analyses that included more frequent or longer sessions [45], indirectly demonstrating that the dose may be a contributing factor to the lower ES of Tuina. The duration of our study was determined based on clinical observations in China. Within our clinical setting, both patients and physicians exhibited the preference for intensive clinical attendance. Nevertheless, further investigation should yield substantiated evidence to explore the relationship of dose-response of Tuina for KOA. Other studies examined that the combination of Tuina with Chinese herbal treatments [22]may benefit from synergistic effects, which could have amplified the overall therapeutic outcomes. It is also important to consider the control group between studies. Previous studies have used cross-over trials, but it can be difficult to establish a sufficient wash-out period to eliminate carry-over effects and to separate the effects of the two treatments, especially when one or both treatments have long-lasting effects [23]. Another study compared Tuina with health education, which also might have resulted in higher ES [22]. The reasons for the disparity in conclusions between our study and others could be attributed to several factors, such as variations in study methodologies and intervention protocols employed across different studies. These discrepancies underline the need for further research to better understand the overall impact of Tuina on KOA.

The potential role of non-specific factors, such as placebo effects and patient expectations, is crucial to consider when evaluating the outcomes of interventions like Tuina and mPT. Both Tuina and mPT involve therapeutic touch and patient-therapist interactions, which can evoke strong placebo effects and shape patient expectations. According to the systematic review, the therapeutic effects of manual therapy may be partly attributed to non-specific factors, rather than solely to the specific physiological or biomechanical actions of the interventions themselves [46]. This highlights the importance of understanding how these non-specific factors may influence treatment outcomes. In the field of manual therapy, the placebo effect has often been undervalued. The tactile characteristics of interventions, coupled with the ritualistic aspects of the therapy, can lead patients to expect positive outcomes, amplifying perceived improvements in pain relief and mobility. It is important to note that while sham control is an essential tool for rigorously assessing the specific effects of an intervention [47], there is a low level of agreement on the appropriate sham procedure for manipulation RCTs [48], and sham groups are not standardized [46]. Therefore, our study did not include a placebo control arm. Although the absence of a placebo control limited our ability to definitively separate the effects of the interventions from the potential influence of placebo and patient expectations, our study directly compared the effectiveness of Tuina with the well-established effectiveness of mPT for KOA, providing a practical and reliable comparison to assess the relative effectiveness of Tuina. To minimize the influence of non-specific factors, our study incorporated psychological assessments of patient expectations to understand how these factors contribute to perceived improvements. The results showed no significant difference in expectations between the two groups, suggesting that observed improvements may not be solely due to differences in expectations. Additionally, we implemented adequate blinding of patients to minimize potential biases related to patient expectations.

There were several strengths in our study. Firstly, this study applied a combination of self-reported subjective and performance-based objective outcomes, to mitigate the potential bias caused by only subjective outcomes, which are more susceptible to the effect of the patient’s expectation or reporting bias (e.g., the wish to please the investigator). Secondly, the blinding assessment results indicated the low risk of unblinding, implied that the patients’ psychological or physical responses less likely to be influenced by knowledge of their treatment status, especially within the context of the TCM hospital. This further allowed patients to make more informed decisions without the influence of preconceived psychological expectations. Additionally, blinding patients to their treatment allocation minimized the risk of measurement bias, thereby providing more reliable and objective results. Thirdly, two therapies exhibited superior safety and only reported local pain symptoms caused by the individual tolerance of manipulation force. Pharmacological treatment, regarded as core management for KOA, was frequently overused and presented an increasing risk of adverse effects [49]. Our results could support the potential for widespread adoption of complementary treatments, thereby contributing to reducing the dependence on pharmacotherapy.

### Study limitations

The limitations of this study included: Firstly, patients in the mPT and Tuina group both received identical information by providing them with the same booklet. This included education about KOA, self-management methods, and simple home-based exercises. The home-based exercises provide by the booklet in both groups were similar to the interventions in the Tuina and mPT groups, which may mask the specific effects of the manual therapies. However, the research team did not have formal tracking. Future studies could exclude routine exercises to avoid bias or implement standardized monitoring protocols for routine exercises. Secondly, this study was conducted at a single center, which may have introduced referral bias and limited the generalizability of the result. Given that our primary objective was to assess the effectiveness of the interventions rather than variations among practitioners, a singlecenter design was chosen for consistency in the delivery of the intervention. Future studies should adopt a multicenter design to enhance the external validity of the findings. Thirdly, the high losses of attrition were noteworthy. The dropout was 8% after treatment, while the rate increased to 44% at follow-up. The reason may be the lack of ongoing treatment or interactions with the practitioners during the follow-up period, which likely reduced patients’ engagement and compliance with evaluations. To assess the robustness of the findings, we conducted a sensitivity analysis using the PP set. Fourthly, future research should aim to include longer follow-up periods to assess the long-term effects of Tuina and mPT interventions. Lastly, there was a lack of information regarding treatment fidelity. Due to the characteristics of manual therapies, some inherent practitioner variability existed in the application of these techniques. This is a recognized challenge in the manual therapies, as the techniques are difficult to standardize and quantify with assessment tools. This inherent challenge highlights the need for more robust fidelity measures in future studies, such as video recordings of treatment sessions or standardized fidelity checklists.

## Conclusions

In conclusion, while no difference was observed between the two groups, it is crucial to note that both interventions yielded clinically relevant improvement in the treatment for KOA. Considering KOA is a progressive disease requiring long-term management, the findings of this trial hold significant potential benefits for patients to have more treatment options available and minimize treatment tolerance.

## Supplementary Information

Supplementary Material 1.

## Data Availability

The deidentified participant data supporting the analyses in the manuscript will be made available upon reasonable written request from researchers whose proposed use of the data for a specific purpose has been approved with publication. For inquiries about data sharing, please send the request to yakno2@163.com.

## Abbreviations

CONSORT: Consolidated Standards of Reporting Trials
ES: Effect size
ITT: Intention-to-treat
KOA: Knee osteoarthritis
MCID: Minimal clinically important difference
mPT: Manual physical therapy
NRS: Numeric rating scale
OMERACT-OARSI: Outcome Measures in Rheumatology Clinical Trials and Osteoarthritis Research Society International
PGA: Patient Global Assessment
PP: Per-protocol set
QoL: Quality of life
SF-12: Short Form Health Survey
TCM: Traditional Chinese medicine
TUG: Timed Up and Go Test Time
Tuina: Tuina therapy
WOMAC: Western Ontario and Mcmaster University Osteoarthritis Index
