# Supplementary material for: Comparative effectiveness of Tuina therapy versus manual physical therapy for knee osteoarthritis: a randomized controlled trial
Source: BMC Complement Med Ther. 2025 Apr 8;25:128. doi: 10.1186/s12906-025-04850-w (PMC11980076; doi:10.1186/s12906-025-04850-w)
Supplement: Supplementary file 1 — Supplementary Material 1. [file 12906_2025_4850_MOESM1_ESM.pdf]

Content of Additional files

Additional file 1 - Original Trial Protocol, and Final Protocol with Amended Changes

Additional file 2 - Complete list of Inclusion Criteria and Exclusion Criteria

Additional file 3 - The response rate defined by the Outcome Measures in Rheumatology Clinical Trials and Osteoarthritis Research Society International (OMERACT-OARSI)

Additional file 4 - Statistical Analysis Plan

Additional file 5 - Baseline Characteristics and Outcome Scores of Participants Who Did and Did Not Complete Primary Outcome

Additional file 6 - WOMAC-total and Sub-Domain Scores, Differences Between Groups, at week 4

Additional file 7- Figure of Secondary Outcomes

Additional file 8 - Participant-Blinding Assessment

Additional file 9 - Matrix Analysis of Tuina and manual Physical Therapy

Additional file 1 - Original Trial Protocol, and Final Protocol with Amended Changes

Comparative Effectiveness of Tuina Therapy Versus Manual Physical Therapy for Knee Osteoarthritis

Context: Original protocol, and final protocol with amended changes (excluding statistical plans)
ClinicalTrials.gov Identifier: NCT03966248
Other Study ID Numbers: DZM-KY201906

Principal Investigator: Xiyou Wang and Changhe Yu

Supported by Beijing University of Chinese Medicine and Capital Clinical Characteristic Application Research (No. Z181100001718165)

Table of contents

Original Protocol (July, 2018 Versions) 4
- **Objective 4**
- **Background 4**
- **Study Design 5**
- **Selection and Enrollment of Patients 7**
- **Assignment of Interventions 9**
- **Study Interventions 9**
- **Outcome Measures 16**
- **Data Management Plan 19**
- **Quality Control 19**
- **Human Participants 20**

Protocol with Amended Changes 21
- **Inclusion Criteria 22**
- **Outcome Measures 22**
- **Blinding 24**

Reference 24

Original Protocol (July, 2018 Versions)

Objective

A single-center parallel active-controlled, blinded randomized controlled trial design was used to verify the effectiveness and safety of Tuina therapy for knee osteoarthritis (KOA). The evidence has shown that manual Physical Therapy (mPT) is effective for KOA. Thus, we hypothesize that the effectiveness of Tuina is different from mPT for the patients with KOA in the changes of the Western Ontario and McMaster Universities Arthritis Index (WOMAC) after 8 treatments at week 4.

Background

1. Osteoarthritis of the knee has become one of the most important topics of medical research nowadays

Osteoarthritis (OA) is a progressive aggravation of the bone and joint, causing joint pain and limited mobility, which seriously affects the quality of life of patients and imposes a huge social and economic burden [1]. KOA is the most commonly involved part of OA, the main symptom of KOA in 48.5% of patients aged 50 years or older is pain [2], and symptomatic KOA is about 2.5 billion [3-4], and it accounts for 10-30% of the world's elderly [5], and has become the fourth most disabling disease in the world [3], with disability loss of life years rising from 10.5 million in 1990 to 17 million in 2010 [6], with the prevalence of KOA reaching approximately 40% in 2025 [7]. The prevalence of KOA in Beijing is approximately 32.2% (42.8% for women and 21.5% for men), with 10.3% of patients presenting with pain (15.0% for women and 5.6% for men) [8]. In the United States, the cost of OA treatment is approximately $3,000 per person per year [9], and medical expenditures rose from $10.3 billion in 2005 to $185.5 billion in 2007 [10]. A study in Beijing showed that KOA patients made an average of 12 hospital visits per year, with an average annual direct cost of $8,858 [11].

2. Current status of KOA treatment recommended by domestic and international guidelines

Currently, domestic and international guidelines emphasize KOA treatment, such as non-pharmacological, non-surgical treatment, and patient education, exercise therapy, and weight control as core therapeutic interventions, are cost-effective and have good long-term outcomes and should be used as core clinical treatments [12-16], Exercise methods include aerobic exercise (e.g., swimming, cycling, etc.), joint function training (knee flexion and extension in the non-weight-bearing position to maintain maximum joint mobility), and muscle strength training (attention should be paid to the abductor muscle groups in the hip joint). Health education and self-management, including explaining the disease regression and the mechanisms of pain through various means such as health lectures, pamphlets, and websites of national organizations, and instructing patients to change their lifestyle habits, exercise patterns, weight control, and other measures, can reduce weight bearing on the degenerated joint. For overweight patients, weight control is recommended to reduce the risk of the disease.

3. Current status of Tuina treatment for KOA

Tuina is used as a complementary alternative medicine in the treatment of pain, especially to relieve pain caused by the musculoskeletal system [17-19], to help relaxation, relieve stress, reduce anxiety and depression levels, and improve quality of life [20-22]. Currently, there are more than 80 massage techniques worldwide, and most foreign clinical and research studies focus on Swedish massage techniques [23], or the variations of massage treatment protocols [24]. Several high-quality randomized control trials (RCTs) have shown their ability to enhance circulation of body fluids, relieve muscle tension and pain, promote metabolism in the body [24], relieve pain, restricted movement, improve quality of life,

and improve emotional problems such as anxiety and depression [25-26].

Although Tuina has existed in traditional Chinese medicine (TCM) for more than 2000 years, domestic clinical practice and research have mostly focused on Tuina techniques for the treatment of related diseases, with widely different techniques used by different schools of thought. Only a few clinical observational studies were exploring the efficacy of Tuina after searching PubMed. Although more clinical trials of Tuina were published in four major Chinese databases, the intervention mostly combined therapy, or with small sample size, poor methodological quality, and publication bias [19,27]. The above situations indicated that there was a lack of credible evidence to prove the clinical effectiveness and safety of Tuina.

The royal TCM-based tendon-regulation manipulation theory originated from the Qing Dynasty, which is one of the major schools of Tuina in TCM. The theory focuses on "light, soft, penetrating, and skillful". The royal TCM-based tendon-regulation manipulation theory has a summary of the physiology and pathology of body movement. The tendons and bones of the human body are tightly connected. When the body moves, the muscles contract to produce force and transfer the force to the bone with the help of tendons or ligaments. At the same time, the bone can efficiently process the force to different parts of ligaments and tendons, resulting in a coordinated motion pattern. Coordination of tendons and bones is the cornerstone of maintaining the dynamic balance of the bones and joints. Tuina manipulation with the therapist's hands can "restrict bone and harmonize joints" to restore the harmonious relationship between tendons and bones.

Based on the theory, the clinical practice and pilot study has shown that the royal TCM-based tendon-regulation manipulation for KOA is effective and safe, but there is a lack of rigorous and normative clinical studies to validate the effectiveness with enough samples. Therefore, we will conduct a trial to compare the effectiveness of Tuina with the recommended manual therapy-mPT for KOA.

Study Design

1. Overview of Study Design

This trial is a single-center parallel active-controlled, blinded randomized controlled trial that aims to compare the effectiveness of Tuina and mPT. A total of 140 qualified KOA patients will be randomly divided into either the Tuina group or mPT group at a ratio of 1: 1. In the Tuina group, patients will receive the Tuina therapy, and patients in the mPT group will receive joint mobilization, soft tissue manipulations, passive stretching, and strength training. They will both receive a total of 8 treatments in a 3-week course. Additionally, both groups will be provided health education and home-exercise instruction. Evaluations will be conducted at baseline (week 0) and 4 and 16 weeks. The primary outcome is the related symptoms of KOA, and secondary outcomes include health-related quality of life, treatment satisfaction, physical limitation, emotional and psychological stress, and adverse events. Figure 1 describes the flow chart of these procedures.

The study consists of 5 phases: recruitment, selection, baseline, treatment, and follow-up.

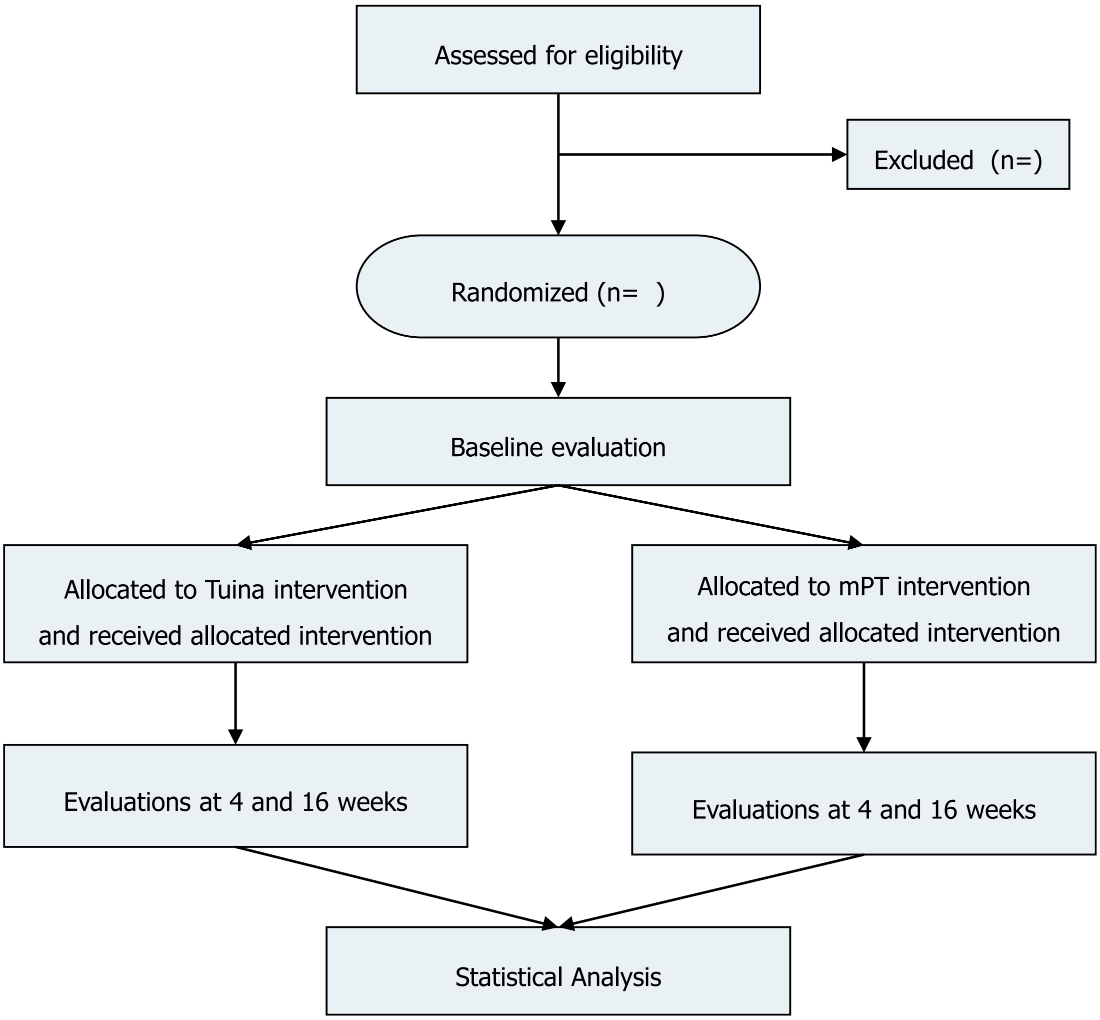

Figure 1 The flow chart

Recruitment: Recruitment will be through multimodal strategies, (posters at the outpatient and community service centers, e-poster online, etc.);

Selection (week -1): Any patients who will be screened will be completing the case screening form. Those who met the screening criteria will be subsequently referred to a specialist for diagnosis and differential diagnosis. Ultimately, the eligible cases were enrolled in the study.

Baseline (week 0): Patients who satisfied the inclusion and exclusion criteria will be identified, randomly allocated, and completed the baseline evaluation period.

Treatment (week 0-4): Patients will be scheduled to be treated by practitioners for 8 sessions over 3 weeks, according to the randomization assignment;

Follow-up (week 5-16): Follow-up assessment will be operated by evaluators.

2. Trial Registration

The protocol will have registered on the website (clinicaltrials.gov: NCT03966248).

3. Study Period

Study design: March, 2018

Approve of Ethical review: Feb 15, 2019

Trial registration and amended: May 26 and 28, 2019

Study Start: October 19, 2019

Study Completion: October, 2021 [Anticipated]

4. The Target Number of Participants and Estimation Basis

(1) Estimation of the number of patients: The following is assumed using the reference literature of prior studies to estimate the number of patients.

(2) Level of significance, αtwo-sides = 0.05.

(3) Type 2 error (β) is set to 0.2, and the power of the test is set to 80%.

(4) Based on our pilot study and the previous studies about the minimal clinically important difference (MCID) [28, 29], we assumed that the primary outcome (a total WOMAC score at 4 weeks) in Tuina group was expected to have mean difference of 7 to mPT with a standard deviation for both groups of 13.

(5) Compliance: 80% (the ratio expected to receive treatment 8 times or more during the 3-week treatment period).

(6) Sample size calculation for the trial by using PASS 15 (Power Analysis and Sample Size Software (2017). NCSS, LLC. Kaysville, Utah, USA, ncss.com/software/pass.)

As a result of applying (1), (2), (3), (4), and effect size, the sample size is calculated to be 140 in total, 70 patients in each group.

Selection and Enrollment of Patients

1. Diagnosis of KOA

Reference to Guidelines for diagnosis and treatment of osteoarthritis (2007 Version) Manufactured by the Chinese Orthopaedic Association [12] and the American College of Rheumatology Arthritis's [30], the diagnosis of osteoarthritis of the knee is as follows (ICD-10code:M17.901):

(1) Knee pain within the last month;

(2) Radio-graphic joint space narrowing, osteophyte(s), subchondral osteosclerosis and/or cystic degeneration shown on a radiograph performed (standing position or weight-bearing position);

(3) > 40 years of age;

(4) Joint fluid examination (at least 2 times) clear, viscous, wbc < 2000/ml;

(5) Morning stiffness≤3 min;

(6) Bone rubbing sound when moving;

The diagnosis of osteoarthritis of the knee should combine clinical, laboratory and radiograph examinations and meeting the criteria of (1)+(2) or (1)+(3)+(5)+(6) or (1)+(4)+(5)+(6).

According to the radiological diagnostic criteria of Kellgren and Lawrecne grade (KL grade), osteoarthritis is classified into five grades.

Grade 0: normal;

Grade I: suspicious narrowing of the joint space and possible osteoarthrosis;

Grade II: significant osteoarthrosis and mild narrowing of the joint space;

Grade III: moderate amount of osteoarthrosis, more definite narrowing of the joint space, mild sclerotic changes in the subchondral bone, and less extensive;

Grade IV: large amount of osteoarthrosis formation, which can spread to the cartilage surface, significant narrowing of the joint space, extremely obvious sclerotic changes, joint hypertrophy and obvious deformity.

2. Inclusion/Exclusion Criteria

Inclusion Criteria:

(1) Diagnosis of KOA;

(2) Age 40-75 years;

(3) KL (Kellgren-Lawrence) grade II or III in recent 6 months;

(4) NRS score of pain at least more than 4 points;
(5) Single/bilateral knee pain, duration of more than 6 month; If the patient has KOA on both legs, the heavier leg is selected for study;
(6) Patients give informed consent, volunteer to be tested and sign an informed consent form

Exclusion Criteria:

(1) The patient has a history of knee surgery or is willing to perform knee surgery (Total Knee Arthroplasty or knee arthroscopy);
(2) Knee pain caused by other diseases (such as joint bodies, the severe effusion of joint cavity, infection, malignant tumors, autoimmune diseases, trauma, etc.);
(3) Oral administration of hormones within 3 months, intra-articular injection within 6 months, knee injuries or open injuries within 6 months, or knee arthroscopy within 1 year;
(4) Severe acute/chronic organic or mental diseases;
(5) Pregnant women, pregnant and lactating women;
(6) Coagulation disorders (such as hemophilia, etc.), or the skin diseases at the site of operation;
(7) Instrument support therapy for patients with knee osteoarthritis disability;
(8) Participation in another clinical study in the past 3 months;
(9) History of receiving acupuncture, massage, manipulation, or any medical plaster treatment within 3 months;
(10) Breaks for treatment longer than 3-4 weeks, depending on each circumstance or merit may be construed as non-compliance and may be excluded.

3. Discontinuation and Dropout Criteria

Study completion status for all patients in the study should be recorded. If the treatment or the follow-up has been discontinued, the discontinuation cause should be recorded. For patients whose study has been discontinued, follow-up can continue with the consent. Cases in which the study can be discontinued for patients who are undergoing the study are as follows:

(1) Depends on the researcher:

- At any time during the study period, if a serious adverse event (Serious Adverse Event, 9.1.) or an adverse event deemed harmful to the patients is observed, the investigator may temporarily suspend this study. Afterward, if it is judged that the treatment continuation adversely affects the participant according to the evaluation of the course of the adverse event and the causal relationship with the treatment, the investigator permanently discontinues the treatment for the patient.
- The patient’s condition deteriorates during the course of the trial, and the proceedings should be stopped according to the physician’s judgment.
- Those who have poor compliance or who withdraw voluntarily in the middle of the trial.
- Other occasions where it is determined that the continuation of the study is not appropriate at the discretion of investigators.

(2) Depends on the patient:

- Patient is unwilling or unable to continue with the clinical trial for whatever reason and discontinues the trial by requesting withdrawal from the trial to the supervising physician;
- Patients is no longer receiving treatment, regardless of whether they explicitly request to withdraw from the trial.

(3) Management of deflated cases

- When a patient is exfoliated, the investigator should take active steps to complete the last test possible for analysis of the effectiveness and safety. For all fall-out cases, case report form (CRF) should be

completed with a trial conclusion form and the reason for the case fall-out. For cases dropped from the trial due to allergic reactions, adverse reactions, or ineffective treatment, the investigator should take appropriate treatment measures according to the actual condition of the patient.

4. Management of Test Suspension

The program is not designed for mid-term analysis and formal discontinuation principles, except for the following:

- A serious safety event occurs in the trial and the trial should be discontinued in a timely manner;
- The trial is found to have a major failure in the clinical trial protocol, or the protocol is good but serious deviations occur in the implementation, the trial should be discontinued;
- the trial found that the treatment effectiveness is poor and does not have clinical value, the trial should be discontinued;
- Administrative authorities to withdraw the trial.

Assignment of Interventions

1. Randomization Sequence Generation

Eligible patients will be randomly assigned to Tuina or PT group in a 1:1 ration. The randomization sequence will be generated using the "Proc plan" program in SAS statistical software (9.3 version).

2. Allocation Concealment Mechanism

Randomization assignment will be concealed using opaque, sealed envelopes sequentially numbered. To ensure concealment, the random sequence will be accessed only by a designated person not involved in participant screening, recruitment scheduling, treatment or assessment. A designated person will prepare the randomization envelopes according to the random sequence, and the envelope is stored separately in a double lock cabinet.

3. Implementation

The patient who signs the written informed consent form and satisfies the inclusion criteria of the study, will be allocated with a sealed randomization envelope. The study coordinator will open the randomization envelope following the sequential order before the participant. Randomization results cannot be viewed in advance and cannot be changed after the assignment.

4. Blinding

Single (Evaluators)

In the initial design, only the evaluators are blinded at the initial stage, and they can't participate in the screening, recruitment scheduling, and treatment. Considering the complicated manipulation of both therapies, we will try to inform patients that they will receive manual therapies, but without detailed information about whether they will receive which kind of therapies. At the end of the trial, the blinding questionnaire will be conducted to assess the success of blindness.

Study Interventions

The practitioners must have a medical certificate and has treated patients with KOA for more than 5 years. Furthermore, they will receive the training of standardized operation procedures before treatment. Both groups receive 20-minutes treatment with 8 sessions over a 3-week treatment period.

Routine Treatment It conduted in both Tuina and mPT groups. Routine treatment includes health education, self-management and home-exercise instruction, and they will be encouraged to perform the simple home-based exercise.

(1) Education and Self-Management

Patients will participate in propaganda about KOA. The context mainly including as below: KOA is a chronic, progressive disease. To mitigate the progression of the disease, patients should avoid risk factors, such as being overweight, prolonged sitting or standing, poor postures, climbing stairs or hills, experiencing joint exposure to cold temperatures, and wearing high-heeled or uncomfortable shoes during exercises. Patients are provided with guidance on the proper usage and adverse reactions of medications.

(2) Home-Exercise Instruction

Squatting Exercise Lean back against a wall and separate the feet to shoulder-width apart. Gradually extend the feet forward to create a distance of approximately 40-50 centimeters from your body's center of gravity, presenting the body with a squatting posture. It is crucial to ensure that the long axis of the lower leg is perpendicular to the ground, with an angle between the thighs and calves of no less than 90 degrees. Generally, each time squat until it is no longer possible to continue, rest for 1-2 minutes, and then repeat. The total number of squats recommended is between 3-6 (depending on the patient's tolerance level). It is best to squat at an angle that does not cause significant pain.

Straight Leg Raise There is no fixed requirement, the weight could be determined according to his strength and feeling. Generally, a weight of 1-3 kg is sufficient. With the leg kept straight, lift the entire leg to create an angle of approximately 30 degrees between the leg and bed, holding for 5 seconds before lowering gradually for 2-3 seconds. It is recommended to complete 3-4 sets of exercises each day. After each set, a rest period of 1-2 minutes is advisable before repeating the next exercise. Effectiveness can be determined by the extent of fatigue felt in the muscles.

Leg tensing Exercise The patient could sit, stand or lie down with their knee straightened. Actively contract the quadriceps, creating tension, while simultaneously ensuring the foot is in full dorsiflexion. Hold for 6 seconds, then relax for 10 seconds, repeat for a total of 10 times.

Stretching Stretching exercises will mainly target the iliopsoas, anterior, posterior, medial and lateral thigh muscles, as well as the posterior, medial, and lateral calf muscle groups. Each muscle group will be stretched for 10-20 seconds.

1. Interventional Group: Tuina

The strength of manipulation should be appropriate to the patients' endurance, and patients received the same assigned practitioner throughout the study to maintain the consistency of manipulations.. The location of acupoints and meridians is described in Table 1. The Tuina therapy mainly includes 6 steps: relaxation, acupoints pressure, patellar manipulation, joint passive movement, strength training and rest.

(1) Soft tissue relaxation (11 minutes)

Soft Tissue Relaxation of the Back of Thigh The patient lies in the prone position, and the practitioner relaxes the posterior side of the affected limb (Bladder meridian) by rolling, kneading, and pushing techniques from the posterior superior iliac spine to the ankle. This process is repeated 5 times.

Soft Tissue Relaxation of the Front of Thigh The patient lies in the supine position, and the practitioner relaxes the anterior (stomach meridian), medial (spleen meridian) and lateral (gallbladder meridian) of the affected limb with the rolling, kneading and pushing methods from anterior superior iliac spine/the highest point of the iliac crest/symphysis pubis to ankle. Additionally, push in the quadriceps, tibialis anterior muscle, from top to bottom by the hand palm root or thenar. This process is repeated 5 times.

Soft Tissue Relaxation of Posterior Knee and Calf The patient lies in the supine position, and the practitioner's fingers in both hands are placed in the gastrocnemius inner and outer head and hamstring muscle, do transverse back and forth. This process is repeated 5 times.

The soft tissue relaxation process takes 11 minutes in total.

(2) Acupoints Pressure (3 minutes)

The patient lies in the supine position, and the practitioner presses the following acupoints: Biguan (ST31), Futu (ST32) by thumb and middle finger of one hand, and the Heding (EX-LE2), Neixiyan (EX-LE4), Waixiyan (Dubi, ST35) by the flexed thumb, forefinger, and middle finger of the other hand simultaneously. And then, the practitioner presses the Zusanli (ST36) by the thumb of one hand and Sanyinjiao (SP6) by the forefinger of the other hand. Finally, the practitioner presses Xuehai (SP10), Liangqiu (ST34), Neixifeng (medial patellofemoral ligament), Waixifeng (lateral patellofemoral ligament), Neixiyan (EX-LE4) and Waixiyan (Dubi, ST35) by the thumbs, forefingers, and middle fingers while two pawls squeezing the knee. One minute for supine position, the process is repeated 3 times in total.

(3) Patellar Manipulation (1.5 minutes)

The patient lies in the supine position, and the practitioner grip the patella with one hand and five fingers, and the other hand assists to fix. Lift the patella up to the maximum extent, so that it leaves the articular surface of the femoral condyle, and then slowly lower it. The operation is repeated 5 times, 0.5 minutes in total. The practitioner presses and kneads on the patella in a clockwise or counterclockwise, for a total of 1 minutes.

(4) Joint Passive Movement (0.5 minute)

The patient lies in supine position, and the practitioner holds the ankle with one hand and the knee joint with the other hand, do maximum flexion and extension with the knee joint, swinging the knee joint quickly 10 times up and down, and then pull the knee joint once. The process is repeated 2 times, 0.5 minute in total.

(5) Strength Training (2 minutes)

Straight Leg Raise The patient lies in supine position, lifts the leg about 0.5 meters off the bed, followed by tightening the quadriceps muscles and keeping the knee locked. After holding the leg for 15-30 seconds, and then lower the leg. The process is repeated 2-3 times, for 1 minute in total.

Cycling in the Air The patient lies in supine position with both knees up to form 90 degere. Crunch up and twist across the body while simultaneously performing a bicycle motion with the legs. The process is repeated for 1 minute.

(6) Rest on Bed (2 minutes)

After the treatment, the patient lies and rests for 2 minutes.

Table 1. Locations of Acupoints and Meridian for Tuina

Acupoints /Meridians	Location
**Biguan (ST31)**	On the anterior aspect of the thigh, on the line connecting the anterior superior iliac spine and the lower lateral border of the patella, level with the lower border of the symphysis pubis, in the depression on the lateral side of sartorius when the thigh is flexed.
**Futu (ST32)**	On the anterolateral aspect of the thigh, on the line connecting the lateral end of the base of the patella with the anterior superior iliac spine, 6 cun superior to the base of the patella
**Heding (EX-LE2)**	On the anterior aspect of the thigh, in the depression superior to the base of the patella
**Zusanli (ST36)**	Three cun directly below ST35, and one finger-breadth lateral to the anterior border of the tibia

Acupoints /Meridians	Location
**Sanyinjiao (SP6)**	On the tibial aspect of the leg, posterior to the medial border of the tibia, 3 cun superior to the prominence of the medial malleolus
**Xuehai (SP10)**	On the anteromedial aspect of the thigh, on the bulge of the vastus medialis muscle, 2 cun superior to the medial end of the base of the patella
**Liangqiu (ST34)**	On the anterolateral aspect of the thigh, between the vastus lateralis muscle and the lateral border of the rectus femoris tendon, 2 cun superior to the base of the patella
**Neixifeng**	On the anterior aspect of the knee, on the medial patellar ligament
**Waixifeng**	On the anterior aspect of the knee, on the lateral patellar ligament
**Neixiyan (EX-LE4)**	On the anterior aspect of the knee, in the depression medial to the patellar ligament
**Waixiyan (Dubi, ST35)**	On the anterior aspect of the knee, in the depression lateral to the patellar ligament
**Spleen meridian on the leg**	The leg part of Spleen meridian starts at the lateral side of the big toe, runs along the medial side of the foot crossing the inner ankle. It then travels along the medial side of the lower leg and thighs reaching the pubic bone.
**Bladder meridian on the leg**	The leg part of bladder meridian runs internally towards the bladder, returns to cross up and down externally on the coccyx region, then continues down the buttock, down the posterior leg towards a point on the medial crease of the posterior knee. The other branch originates from the main branch at the base of the skull and continues downward parallel, but 1 cun more lateral, and continues down until it reaches a point on the middle of the buttock, turns laterally towards a point on the hip, then continues down the posterior leg, crosses through the point on the medial aspect of the posterior knee where the first branch stopped. The two branches join at this point and meridian continues down the back of the lower leg behind the outer ankle, then continues along the outside of the foot and ends on the lateral side of the tip of the little toe
**Gallbladder meridian on the leg**	The leg part of Gallbladder meridian (from lower abdomen and hip) continues to run down the lateral side of the leg, goes over the foot and ends the tip of the fourth toe.
**Stomach meridian on the leg**	The leg part of Stomach meridian crosses the abdomen and groin and goes downward along the front of the thigh and the lower leg until it reaches the top of the foot. Finally, it terminates at the lateral side of the tip of the second toe.

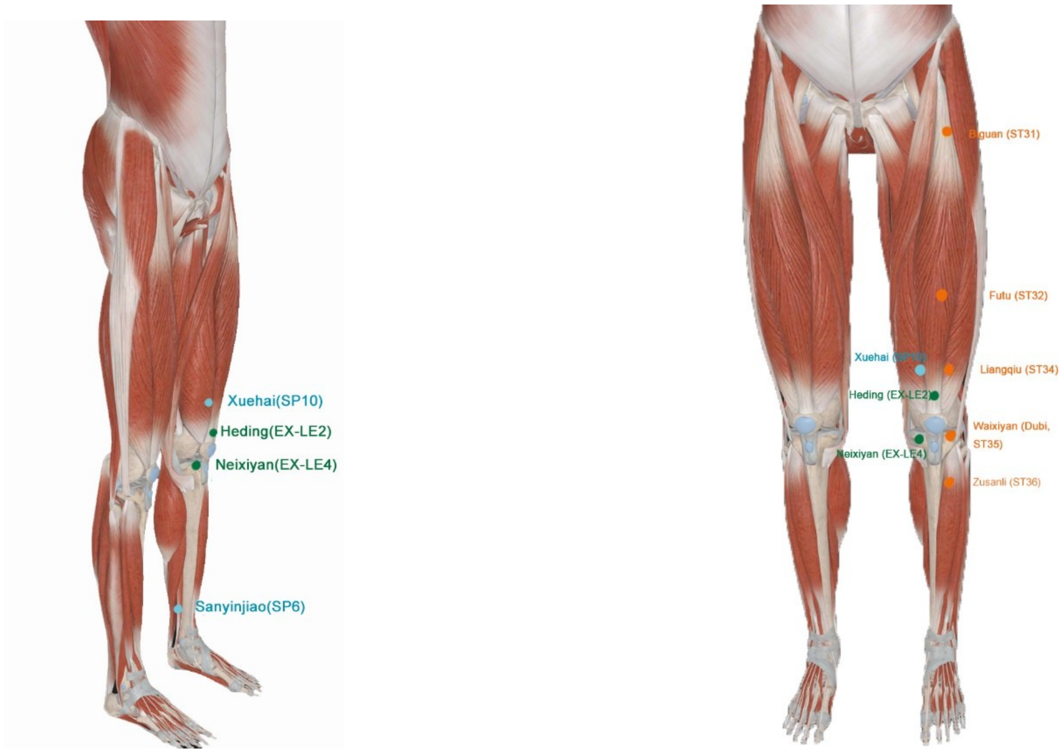

Figure 2. Locations of Acupoints for Tuina

Note: Biguan (ST31), Futu (ST32), Liangqiu (ST34), Waixiyan (Dubi, ST35), Zusanli (ST36); Heding (EX-LE2), Neixiyan (EX-LE4), Xuehai (SP10), Sanyinjiao (SP6). The picture copyright authorization has been obtained from 3Dbody

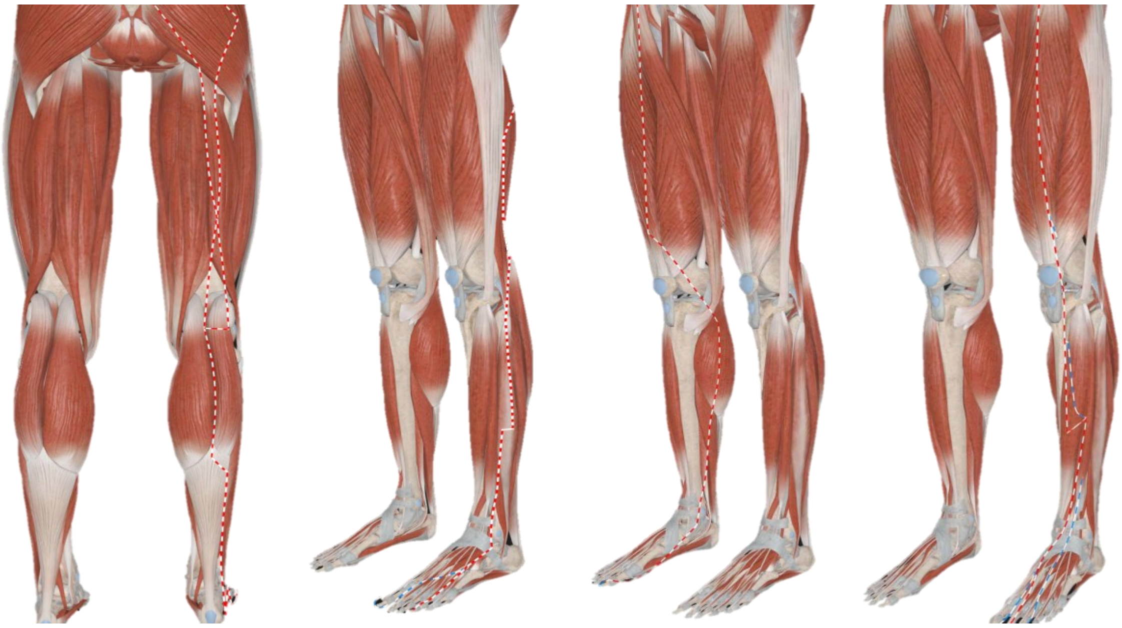

A B C D

Figure 3. Meridian selected in Tuina

Note: A: Bladder meridian on the leg; B: Gallbladder meridian on the leg; C: Spleen meridian on the leg; D: Stomach meridian on the leg. The picture copyright authorization has been obtained from 3Dbody

2. Comparative group: manual Physical Therapy

After enrollment, practitioner will evaluate the initial musculoskeletal status, range of motion. The mPT group mainly includes 5 steps: stretching, soft tissue manual therapy, physiological movements, accessory movements and strength training.

(1) Stretching Manual (3 minutes)

The practitioner stretches the quadriceps, iliopsoas, hamstrings, adductor femoris, abductor femoris (iliotibial band, tensor fascia lata and gluteus muscles) and gastrocnemius.

(2) Soft Tissue Manual (10 minutes)

The practitioner alternates the manual therapy of kneading (using fingers or the palm of the hand to knead the muscles and soft tissues around the knee joint) and pressure (using fingers or the palm of the hand to apply pressure on tender points or tense soft tissues around the knee joint). The above manual therapies are performed on the areas of the quadriceps, hamstrings, adductorfemoris, abductor femoris and gastrocnemius, suprapatellar and peripatellar, medial and lateral capsule, with the requirement that the movements be gentle and slow to avoid causing pain or injury to the patient. The process is repeated 1min/time, with each area being operated 2 minutes.

(3) Grade 3 or 4 Physiological Movements (3 minutes)

Knee Flexion The practitioner stabilizes the patient's thighs and knees and grabs the patient's ankle. The patient's heel is softly moved towards the hip of the restricted movement disorder. And oscillation can be performed in the bending direction. The practitioner could increase both valgus or varus stress and internal or external rotation as needed.

Knee Extension The practitioner places one hand on the patient's patella and the other on the back of their ankle. Keeping their arms straight, the practitioner tilts their body towards the patient's head and lifts the patient's feet off the bed, allowing for mobilization of the knee joint with internal or external rotation or the ankle joint with varus or valgus stress.

Accessory movements (2 minutes)

The definiction of Grade 3 or Grade 4 by Maitland motabilition: Grade 3: Large slow amplitude oscillatory movements performed from the middle to the end of the available joint range. Grade 4: Small slow amplitude movements up to the end of the available joint play.

Femoro-Tibia Articulation Mobilizations (0.5 minutes) Long axis traction: The patient sits in front of the practitioner while the practitioner stands opposite the affected limb. Both knees are flexed to 90°, and the practitioner places one hand on the back of the thigh to match the bent knee while holding the patient's heel with the other hand. The patient's calf is then placed between the practitioner's biceps and shoulder and fixed firmly, while both hands of practitioner are kept fixed at the knee bend. The practitioner tracts the legs downwards towards the floor.

Anterior to Posterior / Posterior to Anterior-Accessory Movements (0.5 Minutes) The patient lies in supine position with their knee flexed and extended as tolerated. The practitioner places their 1st web space on the patient's proximal tibia and applies oscillatory mobilization (Grade 3 or 4) in an anterior-posterior (AP) direction on the proximal tibia-. The manipulation was performed with large slow amplitude oscillatory movements from the middle to the end of the available joint range with Grade 3. If needed, the practitioner can add tibial rotation to effectively reach the restrictive barrier with Grade 4. The practitioner grasps around the proximal tibia and produces an AP movement with their thenar eminence of the lateral hand, while simultaneously producing a posterior-anterior movement with their fingers of the medial hand. This technique results in a lateral rotation of the tibia on the femur.

Medial to Lateral/ Lateral to Medial Accessory Movements (0.5 minutes) The practitioner stabilizes their lateral hand over the distal lateral femur, placing it between their medial forearm and trunk. They place their medial hand on the proximal tibia and apply a lateral push while the lateral hand applies a medial push to the proximal tibia. Then oscillatory mobilization (Grade 3 or 4) is applied in a medial to lateral or lateral to medial direction on the proximal tibia. The manupulation was performed with large slow amplitude oscillatory movements from the middle to the end of the available joint range with Grade 3. If needed, the practitioner can add the small slow amplitude movements up to the end of the available joint play with Grade 4.

Patellar Mobilizations (0.5 minutes) The practitioner stands with their back facing the patient and places the affected leg between their upper arm and trunk, holding the distal leg with both hands. Then they slightly pull the leg down while simultaneously swinging the leg up.

(4) Strength Training (2 minutes)

Straight Leg Raise The patient lies in supine position, lifts the leg about 0.5 meters off the bed, followed by tightening the quadriceps muscles and keeping the knee locked. After holding the leg for 15-30 seconds, and then lower the leg. The process is repeated 2~3 times, for 1 minute in total.

Cycling in the Air The patient lies in supine position with both knees up to form 90°, and keep both hands on the head. Crunch up and twist across the body while simultaneously performing a bicycle motion with the legs. The process is repeated for 1 minute.

(5) Rest on Bed (2 minutes)

4. Attention: Treatment During the Trial

Patients could take medications for KOA if necessary or if taking it for a long time. The treatments such as massage, acupuncture, and other physical therapies, are prohibited during the treatment period. The medications or other treatments for KOA during the trial must be recorded in the CRF.

Outcome Measures

We delay one week after treatment as the first evaluation timepoint (at 4 weeks) to exclude the local pain caused by treatment. Only affected knee will be evaluated from baseline throughout the entire study, if the unilateral osteoarthritis or the heavier knee in cases of bilateral osteoarthritis.

1. Primary Outcome Measure

Change in WOMAC [31]

[Time Frame: Change from baseline at 4 weeks]

Self-report measure of WOMAC total.

2. Secondary Outcome Measure

(1) Change in WOMAC

[Time Frame: Change from baseline at 16 weeks]

Self-report measure of pain, stiffness, and physical function subscale for KOA.

(2) Change in Numeric Rating Scale of Knee Pain [32]

[Time Frame: Change from baseline at 4 weeks and 16 weeks]

Patients will rate their worst knee pain in the last 24 hours using an 11-point numeric pain rating scale with 0 representing "no pain" and 10 representing " the worst pain imaginable".

(3) Change in Patient Global Assessment (PGA) [33]

[Time Frame: Change from baseline at 4 weeks and 16 weeks]

The PGA rating is a 11-point Numeric Rating Scale (NRS) in which patients will rate the degree to which their knee condition has changed from the time treatment was initiated to the present. The participant responds to the following query: "Please rate your overall knee's condition from the time you began treatment until now." using an 11-point NRS with 0 representing "A very great deal better" and 10 representing "A very great deal worse".

(4) OARSI Responder Criteria [34]

[Time Frame: At weeks 4 after randomisation]

Responder criteria included 1) greater than or equal to 50% improvement in WOMAC pain or WOMAC function and an absolute improvement of greater than or equal to 20, or 2) improvement in at least 2 of the following 3 scores: 20% improvement in pain and absolute change ≥ 10 on WOMAC pain score, 20% improvement in pain and absolute change ≥ 10 on WOMAC function score, or moderate or greater improvement (≤ 4) on a 15 point global rating of change scale.

(5) Number of Days With Restricted Activities About KOA

[Time Frame: Change from baseline at 4 weeks]

Restricted activity days, as an interim measure of disability assessment, will be evaluated through three questions regarding KOA. "bedridden at home for half a day or more", "unable to go to work/school", or "elimination of daily activities" due to KOA symptoms over the past week.

(6) Change in 12-item Short Form Health Survey (SF-12) [35]

[Time Frame: Change from baseline at 4 weeks and 16 weeks]

The SF-12 is a generic health-related quality of life measure.

(7) Change in the Mental Health Index (MHI-5) [36]

[Time Frame: Change from baseline at 4 weeks and 16 weeks]

Mental health will be measured by the Mental Health Index (MHI-5), hich consists of a group of five items from the SF-36 survey. The MHI-5 evaluates patients' overall mental health, encompassing depression, anxiety, emotional-behavioral regulation, and general positive mood, and in doing so, correlates strongly with the same composite measure used in the mental health scale.

(8) Change in Perceived Stress Scale (PSS-10) [37]

[Time Frame: Change from baseline at 4 weeks and 16 weeks]

Psychological stress will be measured using the Chinese version of the Perceived Stress Scale (PSS-10). The PSS-10 is a widely utilized self-assessment tool that evaluates perception of stress over the course of approximately one month, and how daily routines, significant events, time assessment, and coping resources influence that experience. Therefore, the PSS is highly sensitive to changes in stress resulting from Tuina intervention.

(9) Change in Tampa Scale for Kinesiophobia [38]

[Time Frame: Change from baseline at 4 weeks and 16 weeks]

The Tampa Scale for Kinesiophobia (TAMPA) is a 17-item scale that provides an important assessment of low back pain fear behaviors. A 4-point Likert scale, with a range from 1 to 4 (strongly disagree, disagree, agree, and strongly agree), was used. Higher scores indicate greater fear of movement. The TAMPA scale exhibits a high level of internal consistency, with a Cronbach alpha value of 0.78, and is also easily modifiable and responsive to variations in treatment.

(10) Blinding Assessment

[Time Frame: At weeks 4 after randomisation]

To test whether the patients are blinded successfully, all patients will be asked to guess which kind of treatment they received.

(11) Credibility/Expectancy Questionnaire [39]

[Time Frame: About 5 min after the first treatment]

The credibility and expectancy of patients will be measured using the Credibility/Expectancy Questionnaire.

(12) Rescue Medicine

[Time Frame: At weeks 4 and 16 after randomization.]

Any use of pain-killers will be ascertained.

3. Other Measures

Adverse events

[Time Frame: At weeks 4 after randomization.]

Table 2. Information collection content and time frame

Measures /Event	Selection/baseline-1st	Week 4-2nd	Week 16-3rd
**Collection time range**	Week -1 or Week 0	Within one week before or after	Within one week before or after
**Informed Consent Form**	X		
**Basic Information**	X		
**WOMAC**	X	X	X
**NRS**	X	X	X
**PGA**		X	X
**OARSI Responder Criteria**		X	
**Number of days with restricted activities**	X	X	
**SF-12**	X	X	X
**MHI-5**	X	X	X
**PSS-10**	X	X	X
**TAMPA**	X	X	X
**Blinding assessment**		X	
**Credibility/Expectancy Questionnaire**	X		
**Rescue medicine**		X	X
**Adverse events**		X	

Data Management Plan

(1) Data Collection

Besides patient specific demographic and personal data (e.g., age, height) medical pre-conditions, previous treatment and previous medication will be documented. Beyond this, during the study, any clinical and treatment related data will be collected (see Table 2. Information collection content and time frame). All data will be retrieved from the CRF.

Information on all cases should be carefully and completely recorded on the CRF, whether the cases are in compliance with the trial protocol or are dislodged cases. The principal investigator shall be responsible for the authenticity of the trial data in the center. CRF annotation and writing of CRFs according to Clinical Data Interchange Standards Consortium (CDISC)'s Study Data Tabulation Model (SDTM) principles.

The data will be meticulously entered by two data entry clerks, ensuring accuracy through a double-entry method. The data entry clerks will be specifically trained with a comprehensive guideline tailored to the study's content. And the data entry process will accordance with the entry guidelines and the specified protocol.

(2) Data Storage

The CRF form and all related original data will be submitted to the hospital where the research group is located for archiving. All cases, whether observed or discharged, should be completed by the requirements of the CRF.

Information Kept by the Study Patients Trial protocol, informed consent form, original reports of serious adverse events, etc.

Information Kept by the Responsible Unit Clinical trial application, preclinical trial information, Ethical materials, randomization schedule, surveillance report, CRF (completed, signed, and dated), serious adverse reaction report, etc.

(3) Data Entry

As described above data will be taken directly from the CRF. Data will be entered to an electronic chart (Microsoft Excel 2010, Microsoft Corp., Redmond, WA, USA) by data entry clerks. All entered data will be cross-checked after entry for correctness. The data will be pseudonymized, processed and stored on PCs and servers of the Dongzhimen Hospital Affiliated to Beijing University of Chinese Medicine. These servers are expertly protected against access over several levels. Access is granted by a person responsible for IT at the hospital, and instructions are issued by the project management.

Quality Control

Rigorous Restriction of Enrollment To control bias, one of the crucial methods utilized involves the rigorous implementation of inclusion criteria. This requires the setting of clear and comprehensive inclusion and exclusion criteria, which restricting the range of eligible patients, minimizing variation among patients, and ultimately supporting the formulation of objective conclusions regarding observed factors.

Protocol Registered and Amend Avoiding selective reporting bias in the publication phase of papers. Register the study protocol online prior to the start of the trial and make changes to the registration site timely in accordance with the progress of the study and changes to the study protocol

Clinical Intervention Before starting the trial, practitioners involved in the trial will be centrally trained with standarised operation procedures, and after training they will be assessed and qualified to participate in the study, and the implementation procedure will also be reviewed regularly.

Evaluation Procedure In order to ensure the consistency of evaluation, evaluator will receive a training to unify the procedure of evaluation. After training, strict assessment should be carried out to check the results of

training. And it is crucial that evaluator will not involve in participant screening, recruitment scheduling and treatment.

The Content of Quality Control Personnel One quality control personnel will be appointed to conduct quality checks through the study. The quality control personnel will conduct regular monitoring visits, plays a crucial role in overseeing the clinical trial.

- Assessing Protocol Adherence: The quality control personnel assesses whether the trial is being conducted according to the approved protocol. They review the procedures to ensure they align with the specified study design, interventions, assessments, and data collection. Any deviations or non-compliance are identified and addressed.

- Reviewing Data Accuracy and Completeness: Informed consent is crucial to ensure participants are fully aware of the trial's purpose, potential risks and benefits, and their rights. The quality control personnel will examine the informed consent process to confirm that participants have given their informed consent voluntarily. They review the consent forms, ensuring they are properly administered, signed, and dated. This helps ensure ethical and legal compliance. Additionally, the quality control personnel conduct thorough checks of the collected data to ensure accuracy and completeness. They review the entered conditions and compare it with the source documents. Discrepancies, errors, or missing information are identified and rectified to maintain data integrity.

Improve the compliance of patients Establishing a WeChat platform to ensure contact with patients, address issues related to KOA in their lives, and reduce discontinuation of cases.

Human Participants

This protocol and the informed consent document and any subsequent modifications will be approved by the Ethics Committee of Dongzhimen Hospital Affiliated to Beijing University of Chinese Medicine (DZMEC-KY-2019-06). The consent form describes the purpose of the study, the procedures to be followed, randomized assignment into one of two groups, and the risks and benefits of participation. The decision regarding participation in the study is entirely voluntary. The patients have their right to retract participation in the study at any time without any penalty, or affecting the quality or quantity of their future medical care, nor loss of benefits to which the participant is otherwise entitled. All questions the patient may have will aim to be answered to the best of their ability by the research assistant. The investigator and the participant should both sign and date the consent form before the person can participate in the study.

(1) Possible risks and management

Both Tuina and mPT should be performed by a professional and clinically experienced practitioner at a designated hospital. Local muscle and soft tissue pain may occur after treatment. To minimize the risk of muscle or joint soreness, the treatment progress will be implemented at interval days. Treatment associated with increased signs and symptoms of pain are suspended until symptoms resolve. If symptoms persist, the participant is referred to the patient's physicians. All patients are informed of any potential risks prior to their participation in any study procedures and are told that they are free to withdraw from the study at any time. Any situations occurred and management during the treatment should be recorded.

(2) Potential Benefits of the Proposed Research

This study is a rigorously designed and implemented effective treatment for osteoarthritis of the knee. The treatment will contribute to relieving pain, improving function, and enhancing quality of life in KOA patients. Patients in the study, regardless of their random assignment, will receive treatment as recommended in current clinical practice or clinical guidelines, and will be treated throughout the whole process.

Protocol with Amended Changes (May 26 and 28, 2019 Versions, registered in the website)

Before registering on the website, the original protocol (2018 version, described as above) was amended, which registered on May 26, 2019. And after registering the website, there was amended again on May 28, 2019. The summary of amended version and parts was summarized in the Table 3.

Table 3. The summary of amended parts

Version	Amend-Before	Amend-After	Reasons
**May 26, 2019 version**	Number of days with restricted activities about KOA, Mental Health Index, Perceived Stress Scale, Tampa Scale for Kinesiophobia	30-second time chair rise test,<br>Timed Up and Go Test Time,<br>One Leg Standing Test,	Considering multiple-dimensions of evaluations of KOA, we changed the outcome questionnaires of activity ability, emotional and psychological stress to some physical performance-based measurements before registering on clinicaltrials.gov.
	Assessment at baseline, week 4, and week 16.	Week 8 assessment of some important outcomes are added	Due to long interval of 4-week and 16-week assessment
**May 28, 2019 version**	KL (Kellgren-Lawrence) grade II or III in recent 6 months	KL (Kellgren-Lawrence) grade II or III in recent 3 months	By shortening the timeframe for recent KL grade II or III, we can capture participants who are currently experiencing active disease and have more recently progressed to the specified grades. This change allows us to focus on homogeneous individuals who are more likely to exhibit ongoing symptoms and functional limitations due to KOA.
	To test whether the participants are blinded successfully, all participants will be asked to guess which kind of acupuncture they received	To test whether the participants are blinded successfully, all participants will be asked to guess which kind of treatment they received	Error in filling in the statement of blinding assessment.
	Single blind (Outcome assessor)	Double blind (Participant, Outcome assessor)<br>Patients only be informed that they received the manual therapies, but on information about whether traditional Tuina or Physical therapy, or no discussion of detailed manual therapy during the trial and treatment. Therapists will not be blinded due to delivering different treatment, but patients, evaluators, and statisticians will be blinded to the group assignment.	In the initial stage of trial, a test of blindness of participants had been conducted. The results showed feasible. The trial determined that participants was blinded.

The revised parts were highlighted in ***bold and Italic font*** as below and the information collection content and time frame showed in the Table 4.

Inclusion Criteria

"KL (Kellgren-Lawrence) grade II or III in recent 6 months" changed to "***KL (Kellgren-Lawrence) grade II or III in recent 3 months***".

Outcome Measures

1. Primary Outcome Measure

Change in Western Ontario and McMaster University Osteoarthritis Index (WOMAC)

[Time Frame: Change from baseline at 4 weeks]

Self-report measure of WOMAC total.

2. Secondary Outcome Measure

(1) Change in WOMAC

[Time Frame: Change from baseline at ***8 weeks*** and 16 weeks]

Self-report measure of pain, stiffness, and physical function subscale for KOA.

(2) Change in Numeric Rating Scale of Knee Pain

[Time Frame: Change from baseline at 4 weeks, ***8 weeks*** and 16 weeks]

Participants will rate their worst knee pain in the last 24 hours using an 11-point numeric pain rating scale with 0 representing "no pain" and 10 representing " the worst pain imaginable".

(3) Change in Patient Global Assessment (PGA)

[Time Frame: Change from baseline at 4 weeks, ***8 weeks*** and 16 weeks]

The PGA rating is a 11-point Numeric Rating Scale (NRS) in which participants will rate the degree to which their knee condition has changed from the time treatment was initiated to the present. The participant responds to the following query: "Please rate your overall knee's condition from the time you began treatment until now." using an 11-point NRS with 0 representing "A very great deal better" and 10 representing "A very great deal worse".

(4) OARSI Responder Criteria

[Time Frame: At weeks 4 after randomization.]

Responder criteria included: 1) greater than or equal to 50% improvement in WOMAC pain or WOMAC function and an absolute improvement of greater than or equal to 20, or 2) improvement in at least 2 of the following 3 scores: 20% improvement in pain and absolute change≥10 on WOMAC pain score, 20% improvement in pain and absolute change ≥ 10 on WOMAC function score, or moderate or greater improvement (≤ 4) on a 15 point global rating of change scale.

(5) Change in 30 second time chair rise test

[Time Frame: Change from baseline at 4 weeks]

Participants will be seated with their arms crossed in front of their chest. On the command "go" participants will stand up and sit down for as many trials as they can in a 30 second period.

(6) Change in Timed Up and Go Test Time

[Time Frame: Change from baseline at 4 weeks]

On the command "go" participants will stand up from a chair, walk 3 meters, turn around, return to the chair and sit down. The time it takes to complete this task will be recorded.

(7) Change in One Leg Standing Test

[Time Frame: Change from baseline at 4 weeks]

One single-leg standing was assessed balance. Test required the subject to stand with arms by his/her side. Timing was started when the subject raised one foot off the ground. Timing was stopped if the subject displaced the weight-bearing foot, touched the suspended foot to the ground, used the suspended limb to support the weight-bearing limb, or reached the maximum balance time of 30 seconds.

(8) Change in 12-item Short Form Health Survey (SF-12)

[Time Frame: Change from baseline at 4 weeks, ***8 weeks*** and 16 weeks]

The SF-12 is a generic health-related quality of life measure.

(9) Blinding assessment

[Time Frame: At weeks 4 after randomisation.]

To test whether the participants are blinded successfully, all participants will be asked to guess which kind of ***treatmen***t they received.

(10) Credibility/Expectancy Questionnaire

[Time Frame: About 5 min after the first treatment]

The credibility and expectancy of participants will be measured using the Credibility/Expectancy Questionnaire.

(11) Rescue medicine

[Time Frame: At weeks 4, 8 and 16 after randomisation.]

Any use of pain-killers will be ascertained.

Table 4. Information collection content and time frame

Measures /Event	Selection /baseline - 1st	Week 4 - 2nd	Week 8 - 3rd	Week 16 - 4th
Collection time range	Week -1 or Week 0	Within one week before or after	Within one week before or after	Within one week before or after
Informed Consent Form	X			
Basic Information	X			
WOMAC	X	X	X	X
NRS	X	X	X	X
PGA	X	X	X	X
OARSI Responder Criteria		X		
Change in 30 second time chair rise test	X	X		
Change in Timed Up and Go Test Time	X	X		
Change in One Leg Standing Test	X	X		
SF-12	X	X	X	X
Blinding assessment		X		
Credibility/Expectancy Questionnaire	X			
Rescue medicine		X	X	X
Adverse events		X		

Blinding

Double (Evaluators and Patients)

During the implementation stage, besides only the evaluators are blinded and they can’t participate in the screening, recruitment scheduling, and treatment. The patients will be informed that they will receive manual therapies, but without detailed information about whether they will receive traditional Tuina or modern mPT. And at the end of the trial, the blinding questionnaire will be conducted to assess the success of blindness. In a short, patients, evaluators and statisticians will be blinded to the group assignment in this trial.

Reference

[1] Disability-adjusted life years (DALYs) for 291 diseases and injuries in 21 regions, 1990–2010: a systematic analysis for the Global Burden of Disease Study 2010[J]. The Lancet, 2012, 380(9859): 2197-2223.

[2] Prevalence of Radiographic Osteoarthritis of the Knee and Its Relationship to Self-Reported Pain[J]. Public Library of Science, 2014, 9(4): e94563.

[3] Years lived with disability (YLDs) for 1160 sequelae of 289 diseases and injuries 1990–2010: a systematic analysis for the Global Burden of Disease Study 2010[J]. The Lancet, 2012, 380(9859): 2163-2196.

[4] Epidemiology of OA[J]. Rheum Dis Clin North Am, 2013, 39(1): 1-1.

[5] Burden of musculoskeletal conditions: osteoarthritis[J]. Best practice and research Clinical rheumatology, 2010, 24: 757-769.

[6] The global burden of hip and knee osteoarthritis: estimates from the Global Burden of Disease 2010 study -- Cross et al. 73(7): 1323 -- Annals of 7the Rheumatic Diseases[J]. Annals of the Rheumatic Diseases, 2014, 73(7): 1323-1330.

[7] Cardiovascular safety of lumiracoxib: a meta-analysis of all randomized controlled trials > or =1 week and up to 1 year in duration of patients with osteoarthritis and rheumatoid arthritis.[J]. Clinical therapeutics, 2005, 27(8): 214-1196.

[8] Comparison of the prevalence of knee osteoarthritis between the elderly Chinese population in Beijing and whites in the United States: The Beijing osteoarthritis study[J]. Arthritis & Rheumatism, 2001, 44(9): 2065-2071.

[9] Direct and Indirect Costs of Pain Therapy for Osteoarthritis in an Insured Population in the United States[J]. Journal of Occupational and Environmental Medicine, 2008, 50(9): 998-1005.

[10] Insurer and out-of-pocket costs of osteoarthritis in the US: Evidence from national survey data[J]. Arthritis & Rheumatism, 2009, 60(12): 3546-3553.

[11] Investigation of medical costs of diseases in patients with knee osteoarthritis[J]. Chinese Medical Journal 2017, 97(1): 29-32.

[12] Guidelines for the diagnosis and treatment of osteoarthritis (2007 edition) [J]. Chinese Journal of Joint Surgery (electronic version)., 2007, 1(4): 281-285.

[13] A consensus statement on the European Society for Clinical and Economic Aspects of Osteoporosis and Osteoarthritis (ESCEO) algorithm for the management of knee osteoarthritis—From evidence-based medicine to the real-life setting[J]. Seminars in Arthritis and Rheumatism, 2016, 45(4): S3-S11.

[14] Care and management of osteoarthritis in adults: summary of NICE guidance[J]. BMJ, 2008, 336(7642): 502-503.

[15] Treatment of Osteoarthritis of the Knee: Evidence-Based Guideline, 2nd Edition[J]. Journal of the American Academy of Orthopaedic Surgeons, 2013, 821(9): 571-576.

[16] OARSI guidelines for the non-surgical management of knee osteoarthritis[J]. Osteoarthritis Cartilage, 2014, 22(3): 363-388.

[17] Massage therapy for osteoarthritis of the knee: a randomized controlled trial.[J]. Archives of internal medicine, 2006, 166(22): 8-2533.

[18] A review of the evidence for the effectiveness, safety, and cost of acupuncture, massage therapy, and spinal manipulation for back pain[J]. Acupuncture in Medicine, 2003, 21(4): 158-159.

[19] Recommendations for the treatment of osteoarthritis based on evidence-based medicine[J]. Chinese Journal of Joint Surgery (electronic version), 2013, vol. missing(3). 397-403.

[20] Massage for Mechanical Neck Disorders: A Systematic Review[J]. Spine, 2007, 32(3): 353-362.

[21] Effectiveness of therapeutic massage for generalized anxiety disorder: a randomized controlled trial.[J]. Depression and anxiety, 2010, 27(5): 50-441.

[22] Massage therapy has short-term benefits for people with common musculoskeletal disorders compared to no treatment: a systematic review[J]. J Physiother, 2015, 61(3): 106-116.

[23] Evidence-based Evaluation of Complementary Health Approaches for Pain Management in the United States[J]. Mayo Clinic Proceedings, 2016, 91(9): 1292-1306.

[24] Swedish Massage: A Systematic Review of its Physical and Psychological Benefits[J]. Advances in mind-body medicine, 2017, 31(2): 16.

[25] Efficacy of physiotherapy management of knee joint osteoarthritis: a randomised, double blind, placebo controlled trial.[J]. Annals of the rheumatic diseases, 2005, 64(6): 12-906.

[26] Physical therapy interventions for knee pain secondary to osteoarthritis: a systematic review.[J]. Annals of internal medicine, 2012, 157(9): 44-632.9

[27] A reticulated Meta-analysis of Chinese medicine dialectical treatment of knee osteoarthritis[J]. Chinese Journal of Evidence-Based Medicine, 2016(5): 532-542.

[28] Bellamy N, Hochberg M, Tubach F, Martin-Mola E, Awada H, Bombardier C, et al. Development of multinational definitions of minimal clinically important improvement and patient acceptable symptomatic state in osteoarthritis. Arthritis Care Res (Hoboken). 2015;67(7):972-80.

[29] Wang Xiyou GY, Li Yang, Jiang Hao, Liu Changxin. The clinical observation of Sun's tuina with nine steps taking 8 minutes for 30 KOA patients (Chinese version). Beijing Journal of Traditional Chinese Medicine. 2014;33(07):522-5.

[30] Altman R, Asch E, Bloch D, Bole G, Borenstein D, Brandt K, Christy W, Cooke TD, Greenwald R, Hochberg M, et al. Development of criteria for the classification and reporting of osteoarthritis. Classification of osteoarthritis of the knee. Diagnostic and Therapeutic Criteria Committee of the American Rheumatism Association. Arthritis Rheum. 1986 Aug;29(8):1039-49. doi: 10.1002/art.1780290816. PMID: 3741515.

[31]Bellamy N, Buchanan WW, Goldsmith CH, Campbell J, Stitt LW. Validation study of WOMAC: a health status instrument for measuring clinically important patient relevant outcomes to antirheumatic drug therapy in patients with osteoarthritis of the hip or knee. J Rheumatol. 1988 Dec;15(12):1833-40. PMID: 3068365.

[32]Farrar JT, Young JP Jr, LaMoreaux L, Werth JL, Poole MR. Clinical importance of changes in chronic pain intensity measured on an 11-point numerical pain rating scale. Pain. 2001 Nov;94(2):149-158. doi: 10.1016/S0304-3959(01)00349-9. PMID: 11690728.

[33] Rohekar G, Pope J. Test-retest reliability of patient global assessment and physician global assessment in rheumatoid arthritis. J Rheumatol. 2009 Oct;36(10):2178-82. doi: 10.3899/jrheum.090084. Epub 2009 Sep 15. PMID: 19755617.

[34] Pham T, Van Der Heijde D, Lassere M, Altman RD, Anderson JJ, Bellamy N, Hochberg M, Simon L, Strand V, Woodworth T, Dougados M; OMERACT-OARSI. Outcome variables for osteoarthritis clinical trials: The OMERACT-OARSI set of responder criteria. J Rheumatol. 2003 Jul;30(7):1648-54. PMID: 12858473.

[35] Gandek B, Ware JE, Aaronson NK, Apolone G, Bjorner JB, Brazier JE, Bullinger M, Kaasa S, Leplege A, Prieto L, Sullivan M. Cross-validation of item selection and scoring for the SF-12 Health Survey in nine countries: results from the IQOLA Project. International Quality of Life Assessment. J Clin Epidemiol. 1998 Nov;51(11):1171-8. doi: 10.1016/s0895-4356(98)00109-7. PMID: 9817135.

[36] Strand BH, Dalgard OS, Tambs K, Rognerud M. Measuring the mental health status of the Norwegian population: a comparison of the instruments SCL-25, SCL-10, SCL-5 and MHI-5 (SF-36). Nord J Psychiatry. 2003;57(2):113-8. doi: 10.1080/08039480310000932. PMID: 12745773.

[37] Sun Y, Gao L, Kan Y, Shi BX. The Perceived Stress Scale-10 (PSS-10) is reliable and has construct validity in Chinese patients with systemic lupus erythematosus. Lupus. 2019 Feb;28(2):149-155. doi: 10.1177/0961203318815595. Epub 2018 Dec 5. PMID: 30518288.

[38] Wong WS, Kwok HY, Luk KD, Chow YF, Mak KH, Tam BK, Wong ET, Fielding R. Fear of movement/(re)injury in Chinese patients with chronic pain: Factorial validity of the Chinese version of the Tampa Scale for Kinesiophobia. J Rehabil Med. 2010 Jul;42(7):620-9. doi: 10.2340/16501977-0575. PMID: 20603691.

[39]Devilly GJ, Borkovec TD. Psychometric properties of the credibility/expectancy questionnaire. J Behav Ther Exp Psychiatry. 2000 Jun;31(2):73-86. doi: 10.1016/s0005-7916(00)00012-4. PMID: 11132119.

Additional file 2-Complete list of Inclusion Criteria and Exclusion Criteria

Inclusion Criteria

(1) radiologic confirmation of osteoarthritis (Kellgren-Lawrence score II or III) in recent 3 months,

(2) a pain score of at least 4 points on the numeric rating scale (NRS) (range:0-10; higher scores indicate greater pain),

(3) single/bilateral knee pain, and a duration of knee joint pain at least 6 months.

Exclusion Criteria

(1) The patient has a history of knee surgery or is willing to perform knee surgery (Total Knee Arthroplasty or knee arthroscopy);

(2) Knee pain caused by other diseases (such as joint bodies, the severe effusion of joint cavity, infection, malignant tumors, autoimmune diseases, trauma, etc.);

(3) Oral administration of hormones within 3 months, intra-articular injection within 6 months, knee injuries or open injuries within 6 months, or knee arthroscopy within 1 year;

(4) Severe acute/chronic organic or mental diseases;

(5) Pregnant women, pregnant and lactating women;

(6) Coagulation disorders (such as hemophilia, etc.), or the skin diseases at the site of operation;

(7) Instrument support therapy for patients with knee osteoarthritis disability;

(8) Participation in another clinical study in the past 3 months;

(9) History of receiving acupuncture, massage, manipulation, or any medical plaster treatment within 3 months;

(10) Breaks for treatment longer than 3-4 weeks, depending on each circumstance or merit may be construed as non-compliance and may be excluded.

Additional file 3- The response rate defined by the Outcome Measures in Rheumatology Clinical Trials and Osteoarthritis Research Society International (OMERACT-OARSI)

The response rate was defined as the proportion of patients who achieved the following criteria at week 4: 1) ≥ 50% improvement in WOMAC-pain or WOMAC-function and an absolute improvement of ≥ 20, or 2) improvement in at least 2 of the following 3: 20% improvement in pain and absolute change ≥ 10 on WOMAC-pain, 20% improvem ent in pain; absolute change ≥ 10 on WOMAC-function; or moderate or greater improvement (≤ 4).

Additional file 4-Statistical Analysis Plan

1. Trial Design

This is a single-center parallel active-controlled, blinded randomized controlled trial of comparing Chinese Tuina therapy and manual physical therapy (mPT) for patients with KOA. This trial aims to validate the effectiveness and safety of Tuina for KOA compared with mPT.

2. Timing of outcome assessments

Outcomes are collected at baseline, week 4, week 8 and week 16.

3. Interim analyses

There are no interim analyses in this study.

4. Level of statistical significance and Confidence intervals to be reported

All applicable statistical tests will be 2-sided and will be performed using a 5% significance level. All confidence intervals will be 95% confidence intervals.

5. Analysis Populations

The primary analysis will be based on the principle of intention-to-treat (ITT), defined as all available data from all randomized patients were originally assigned, regardless of their adherence to their assigned treatments.

6. Data of Trial Population

Screening Data Screening data will be collected and summarized in text and/or flow diagram, the following summaries will be presented as below: the recruitment timeline, the number of participants assessed for eligibility screened, the number of participants recruited, the number of participants excluded, and the reasons for exclusion.

Recruitment The number of people enrolled, randomized, allocated to each treatment group, lost to follow up (including reasons) and analyses will be presented in the flow diagram.

Baseline Characteristics Baseline characteristics will be summarised and presented in a table:

-Age, Sex, BMI (kg/m^2), Race

-Duration (Months), Kellgren criteria, Target knees

-Education, Working status, Marital status

-Concomitant disease

-History of treatment, the Satisfaction of Previous Treatment

-WOMAC, NRS, SF-12

-30 second time chair rise test, Timed Up and Go Test Time, One Leg Standing Test

-Credibility/Expectancy Questionnaire

Baseline characteristics will be summarized as appropriate (means and standard deviations for continuous variables for normal distribution, medians and interquartile ranges for abnormal distribution, counts and percentages for categorical variables). Tests of statistical significance will not be undertaken for comparing baseline characteristics of treatment groups.

7. Outcome Definitions

Primary Outcome The change in the total Western Ontario and McMaster University Osteoarthritis Index (WOMAC) from baseline to 4 weeks

Secondary Outcomes The secondary outcomes covered pain, physical function, and patients' overall assessment of disease severity.

- **Pain Dimensions** WOMAC pain (the change between the baseline and 4, 8, and 16 weeks), the worst knee pain in the last 24 hour using NRS(the change between the baseline and 4, 8, and 16 weeks) ;

- **Function Dimensions** the average function of previous week using WOMAC function (the change between the baseline and 4, 8, and 16 weeks),WOMAC stiffness(the change between the baseline and 4, 8, and 16 weeks), 30 Second Time Chair Rise Test (the change between the baseline and week 4), Timed Up and Go Test Time (the change between the baseline and week 4), One Leg Standing Test (the change between the baseline and week 4);
- **Patients' overall assessment of disease severity:** the 12-item Short Form Health Survey (the change between the baseline and 4, 8, and 16 weeks), the patient global assessment (the change between 4, 8, and 16 weeks);
- **Criteria of Response Rate:** The OMERACT-OARSI responder criteria to assess the response rate of treatment for KOA at 4 weeks.
- **The blinding assessment**

8. Analysis methods

We will use intention-to-treat analyses, analyses will be conducted by a statistician blinded to treatment allocation, with two-sided hypothesis tests. Changes from baseline will be presented for each group at each time point using the mean change and 95% confidence intervals.

Primary outcome Primary outcome will apply the repeated measures analysis of variance. Age, sex, BMI, baseline WOMAC score, medication as the covariates. The mean difference in change from baseline to 4 weeks of WOMAC total as the dependent variables, and group (Tuina group and mPT), time (baseline, week 4,8 and 16), and the group × time interaction as independent variables. The approximate normality and Mauchly's test of sphericity were examined. Then the interaction of group-interaction term was tested first. If significant, between-group differences at each time point were tested. If not significant, the treatment main effect was tested next.

Secondary outcomes For repeated measures of continuous outcome, including the subscale of WOMAC, NRS, SF-12 and PGA, will be estimated using the repeated measures analysis of variance, 30 Second Time Chair Rise Test, TUG and One Leg Standing Test will be applied with an independent t test for normal distribution, Mann-Whitney U test for unnormal distribution. And for categorical variables- responder criteria, will be compared across groups with the $\chi 2$ tests or Fisher's exact test.

Blinding outcome Blinding assessment will apply the James blinding index with the blinding package of SAS software.

Effect size Effect size will be computed with mean group-difference/baseline SD; **MCID** The minimal clinically important difference (MCID) of WOMAC- total is set as 14%, which will be calculated with the change of baseline and the corresponding evaluation time point/baseline

9. Statistical Methods – Sensitivity analyses

A sensitivity analysis will estimate treatment effects on the primary outcome at 4 weeks assuming only patients who completed the treatment without major protocol violations at week 4. The sensitivity analysis will be based on t-tests for continuous variables and chi-squared tests for categorical variables

10. Missing data reporting and assumptions/statistical methods to handle missing data

Missing data will be handled with the multiple imputation with 20 iterations for the primary outcome. Baseline characteristics of patients who provided the primary outcome and those who did not will be compared using t tests or chi-square tests.

11. Statistical Software

SPSS 24.0 (SPSS Inc.) will be mainly used for calculations in this study.

Additional file 5 - Baseline Characteristics and Outcome Scores of Participants Who Did and Did Not Complete Primary Outcome

Characteristics	Missing primary outcome (n =13) [a]	Provided primary outcome (n=127) [b]	*P* value
**Age, mean (SD), years**	57.38 (9.2)	60.35 (7.77)	0.199
**Female, No. (%)**	13 (100.00%)	96 (88.07%)	0.095
**BMI, mean (SD), kg/m^2**	24.35 (2.24)	25.09 (3.05)	
**Race, n (%)**			
Han	10 (76.92%)	122 (96.06%)	0.027
Minorities	3 (23.08%)	5 (3.93%)	
**Duration, Median (IQR), Months**	41.00 (9.50,114.50)	44.00 (10.75,109.75)	0.691
**Kellgren criteria, No. (%)**			
Kellgren II	6 (46.15%)	71 (55.90%)	0.50
Kellgren III	7 (53.84%)	56 (44.09%)	
**Concomitant disease, No. (%)**			
0	7 (53.85%)	54 (42.52%)	0.540
1	5 (38.46%)	41 (32.28%)	
2	1 (7.69%)	23 (18.11%)	
≥3	0 (0%)	9 (7.09%)	
**Education, No. (%)**			
High school or less	12 (92.30%)	82 (64.57%)	0.124
College	1 (7.69%)	36 (28.35%)	
Advanced degree	0 (0%)	9 (7.09%)	
**Working status, No. (%)**			
Employed	5 (38.46%)	65 (51.18%)	0.339
Retired	8 (61.54%)	54 (42.52%)	
Others	0 (0%)	8 (6.30%)	
**Marital status, No. (%)**			
Married	10 (76.92%)	117 (92.12%)	0.194
**History of treatment, No. (%)**			
Pharmaceuticals	11 (84.62%)	108 (85.04%)	1.000
Intervention therapy	5 (38.46%)	66 (51.97%)	0.353
Physiotherapy	6 (46.15%)	76 (59.84%)	0.340
Others	0 (0%)	10 (7.88%)	0.628
**The Satisfaction of Previous Treatment, mean (SD)**	5.50 (1.99)	5.25 (2.00)	0.533
**WOMAC, mean (SD)**			
Total	18.85 (11.34)	19.26 (13.51)	0.915
Pain subscale	4.15 (2.38)	4.57 (2.88)	0.619
Stiffness subscale	1.31 (1.44)	1.57 (1.74)	0.605
Function subscale	13.38 (8.39)	13.13 (10.06)	0.929
**NRS, mean (SD)**	5.50 (1.43)	5.90 (1.55)	0.378

**SF-12, mean (SD)**			
Physical health	37.41 (8.34)	37.85 (7.55)	0.746
Mental health	48.23 (10.02)	51.39 (9.41)	0.057
**30 Second Time Chair Rise Test, mean (SD), times**	10.08 (3.98)	10.96 (3.62)	0.667
**Timed Up and Go Test Time, mean (SD), second**	11.24 (1.84)	11.30 (2.96)	0.906
**One Leg Standing Test, mean (SD), second**	21.95 (11.36)	18.76 (10.63)	0.928
**Target knees, No. (%)**			
Left	6 (46.15%)	60 (47.24%)	0.940
Right	7 (53.85%)	67 (52.76%)	
**Credibility/Expectancy Questionnaire, mean (SD)**			
Credibility questionnaire	-0.17 (-0.70,0.47)	0.28 (-0.40,0.74)	0.20
Expectancy questionnaire	0.59 (-0.79,0.72)	0.13 (-0.78,0.58)	0.96

Note: [a]: Seven patients in the Tuina group, 6 in the mPT group, did not complete the primary outcome – WOMAC-total at week 4. [b]: 63 patients in the Tuina group, 64 in the mPT group, complete the primary outcome – WOMAC-total at week 4. *p*-values based on t-tests for continuous characteristics and chi-squared tests for categorical characteristics.

Additional file 6 - WOMAC-total and Sub-Domain Scores, Differences Between Groups, at week 4 (Per Protocol set)

Outcomes	Tuina (n=63)	mPT (n=64)	Between-group comparison Mean difference (95% CI)	*P* value
**Primary outcome**				
**WOMAC-Total, mean (SD)**				
Baseline	19.10 (13.52)	19.42 (13.62)	-0.33 (-5.09, 4.44)	0.892
Week 4	13.44 (12.46	13.58 (12.70)	-0.13 (-4.55, 4.29)	0.952
Mean change from baseline-week 4 (95% CI)	-5.65 (-8.33, -2.97)	-5.84 (-9.56, -2.12)	0.19 (-4.36, 4.74)	0.933
**WOMAC-Pain, mean (SD)**				
Baseline	4.49 (2.80)	4.64 (2.98)	-0.15 (-1.17,0.87)	0.773
Week 4	2.75 (2.38)	3.06 (2.62)	-0.32 (-1.20,0.56)	0.478
Mean change from baseline-week 4 (95% CI)	-1.75 (-2.44, -1.05)	-1.58 (-2.37, -0.78)	-0.17 (-1.21, 0.88)	0.751
**WOMAC-Stiffness, mean (SD)**				
Baseline	1.71 (1.88)	1.42 (1.60)	0.29 (-0.32, 0.91)	0.347
Week 4	1.37 (1.61)	1.12 (1.44)	0.24 (-0.30, 0.78)	0.377
Mean change from baseline-week 4 (95% CI)	-0.35 (-0.71, 0.01)	-0.30 (-0.71, 0.12)	-0.05 (-0.60, 0.49)	0.850
**WOMAC-Function, mean (SD)**				
Baseline	12.89 (10.00)	13.36 (10.19)	-0.47 (-4.02, -3.08)	0.793
Week 4	9.30 (9.32)	9.39 (9.37)	-0.09 (-3.37, 3.19)	0.957
Mean change from baseline-week 4 (95% CI)	-3.59 (-5.54, -1.64)	-3.97 (-6.74, -1.19)	0.38 (-2.99, 3.75)	0.823

Additional file 7 - Figures of Secondary Outcomes

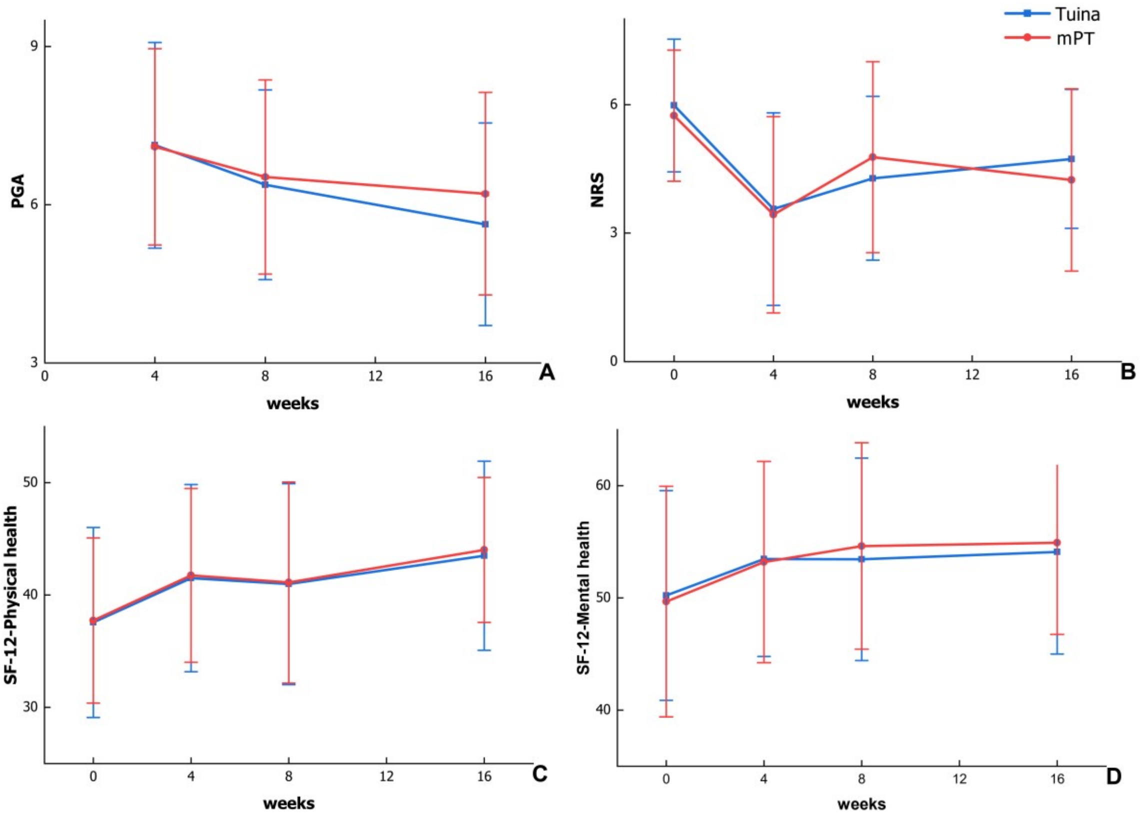

Figure. The secondary outcomes change over time

Abbre: mPT: manual Physical Therapy; NRS: Numeric Rating Scale; PGA: the Patient Global Assessment; SF-12: 12-item Short Form Health Survey, 95%CI :95%Confidence Interval

Note: Data presented as mean and 95%CI. Blue line: Tuina group; Red line: Manual Physical Therapy group; A: The NRS change over time; B: The PGA change over time; C: The SF-12 Physical health change over time; D: The SF-12 Mental health change over time.

Additional file 8 - Participant-Blinding Assessment

Treatment guess, n (%) / Group (Week 4)	Tuina	mPT	Unsure
**Tuina (n =63) [a]**	19 (30.16%)	6 (9.52%)	38 (60.32%)
**mPT (n=64) [a]**	14 (21.88%)	13 (20.31%)	37 (57.81%)
**James blinding Index [b]**	0.75 (95%CI, 0.68 to 0.81)		

Abbreviation: mPT=manual Physical Therapy

Note: [a]: Seven patients in the Tuina group, and 6 in the mPT group, did not complete the blinding assessment at week 4. [b]: James blinding index range: 0 indicates the total absence of blinding, 1 indicates complete blinding, and 0.5 indicates completely random blinding.

Additional file 9 - Matrix Analysis of Tuina and Manual Physical Therapy

Group	Interven-tions*	Muscle and tendons							Joints			Purpose#
		Thigh				Calf						
		Anterior	Posterior	Medial	Lateral	Anterior	Posterior	Lateral	Popli-teal fossa	Periprosthetic tissue	Knee	
Tuina	①	√	√	√	√		√		√	√	√	1,2,3,4
	②	√		√	√	√		√			√	2,3
	③									√	√	2,3,5
	④	√	√	√	√	√	√	√	√	√	√	5
	⑤	√	√	√	√	√	√	√	√		√	4, 5, 6
	⑥											
mPT	①	√	√	√	√	√	√		√√			1
	②	√	√	√	√		√	√	√	√	√	2, 3
	③	√	√	√	√	√		√			√	1,5
	④	√	√	√	√	√	√	√	√	√	√	2,3,4,5,6
	⑤	√	√	√	√	√	√	√	√		√	4, 5, 6
	⑥											

Note: *Interventions:

Tuina: ① Soft tissue relaxation of lower limbs (11 minutes), ② Acupoints pressure (3 minutes), ③ Patellar manipulation (1.5 minutes), ④ Joint passive movement (0.5 minutes), ⑤ Strength training (2 minutes), ⑥ Rest on bed (2 minutes).

mPT: ① Stretching Manual (3 minutes), ② Soft tissue Manual (10 minutes), ③ Grade 3 or 4 Physiological movements (1 minutes), ④ Accessory movements (2 minutes), ⑤ Strength training (2 minutes), ⑥ Rest on bed (2 minutes)

Purpose: 1. improve soft tissue extension; 2. soft local tissues; 3. reduce soft tissue swelling, adhesions or inflammation; 4. change muscle function; 5. increase joint mobility; 6. improve joint stability.

Reference

Si-na LI, Xi-you WANG, Jing-quan TANG, Duo-duo LI, Chang-xin LIU, Chang-he YU. Standardization study on the different types of tuina treatments for knee osteoarthritis in the clinical trials. China Journal of Traditional Chinese Medicine and Pharmacy. 2020, 35 (3): 1422-1427
